# Health effects of saturated and trans-fatty acid intake in children and adolescents: Systematic review and meta-analysis

**DOI:** 10.1371/journal.pone.0186672

**Published:** 2017-11-17

**Authors:** Lisa Te Morenga, Jason M. Montez

**Affiliations:** 1 Department of Human Nutrition, Edgar Diabetes and Research Centre, and Riddet Institute, University of Otago, Dunedin, New Zealand; 2 Department of Nutrition for Health and Development, World Health Organization, Geneva, Switzerland; CUNY, UNITED STATES

## Abstract

**Background:**

Elevated cholesterol has been linked to cardiovascular disease in adults and preclinical markers of atherosclerosis in children, thus reducing saturated (SFA) and trans-fatty acids (TFA) intake from an early age may help to reduce cholesterol and the risk of cardiovascular disease later in life. The aim of this review is to examine the evidence for health effects associated with reducing SFA and TFA intake in free-living children, adolescents and young adults between 2 to 19 years of age.

**Design:**

Systematic review and meta-analysis of randomised controlled trials (RCTs) and prospective cohort studies. Study selection, assessment, validity, data extraction, and analysis were undertaken as specified by the Cochrane Collaboration and the GRADE working group. Data were pooled using inverse variance models with random effects.

**Data sources:**

EMBASE; PubMed; Cochrane Central Register of Controlled Trials; LILACS; and WHO Clinical Trial Registry (up to July 2016).

**Eligibility criteria for selecting trials:**

RCTs involving dietary interventions aiming to reduce SFA or TFA intakes and a control group, and cohort studies reporting the effects of SFA or TFA exposures, on outcomes including blood lipids; measures of growth; blood pressure; insulin resistance; and potential adverse effects. Minimum duration was 13 days for RCTs and one year for cohort studies. Trials of weight loss or confounded by additional medical or lifestyle interventions were excluded.

**Results:**

Compared with control diets, there was a highly statistically significant effect of reduced SFA intake on total cholesterol (mean difference (MD) -0.16 mmol/l, [95% confidence interval (CI): -0.25 to -0.07]), LDL cholesterol (MD -0.13 mmol/l [95% CI:-0.22 to -0.03]) and diastolic blood pressure (MD -1.45 mmol/l [95% CI:-2.34 to -0.56]). There were no significant effects on any other risk factors and no evidence of adverse effects.

**Conclusions:**

Advice to reduce saturated fatty acids intake of children results in a significant reduction in total and LDL-cholesterol levels as well as diastolic blood pressure without evidence of adverse effects on growth and development. Dietary guidelines for children and adolescents should continue to recommend diets low in saturated fat.

## Introduction

Dietary saturated fatty acids (SFA) and trans-fatty acids (TFA) are strongly correlated with total and low-density lipoprotein (LDL) cholesterol levels in adults[1–4], both well-established markers of cardiovascular disease (CVD).[5, 6] Reduced intakes of SFA have been shown to be are associated with significant reduction in risk of CVD, particularly when replaced by polyunsaturated fatty acids (PUFA) in both randomised trials and cohort studies.[7–10] Similarly, cohort studies demonstrate that high intakes of TFA are strongly associated with increased likelihood of coronary heart disease and related mortality.[11]

Although cardiovascular and coronary heart diseases typically present later in life, atherosclerotic lesions in the aorta and coronary arteries can begin to appear in childhood, [12, 13] and are positively associated with dyslipidaemia and other CVD risk factors.[13, 14] Elevated total and LDL cholesterol in childhood are, in turn, associated with an increase in CVD risk factors in adulthood[14] including thickening of the carotid artery intima-media,[15–17] a marker of subclinical atherosclerosis and predictor of future cardiovascular Dietary saturated fatty acids (SFA) and trans-fatty acids (TFA) are strongly correlated with total and low-density lipoprotein (LDL) cholesterol levels in adults[1–4], both well-established markers of cardiovascular disease (CVD).[5, 6] Reduced intakes of SFA have been shown to be associated with significant reduction in risk of CVD in meta-analyses of both prospective cohort studies and randomised controlled trials (RCTs), with the strongest associations seen when SFA are replaced by polyunsaturated fatty acids (PUFA).[7–10] Similarly, cohort studies demonstrate that high intakes of TFA are strongly associated with increased likelihood of coronary heart disease and related mortality.[11] Controversially a recent meta-analysis of RCTs incorporating recovered and reanalysed data from two studies conducted fifty years ago [18, 19] suggests increased risk of coronary heart disease death and cardiovascular disease when replacing SFA with n-6 PUFA.[18] The limitations of this new analysis have been widely reported.[20, 21]

The World Health Organization (WHO) is currently updating its guidance on SFA and TFA intake in adults and children. The aim of this systematic review and meta-analysis is to examine the evidence for health effects associated with reducing SFA and TFA intake in children in order to inform and contribute to the development of updated WHO recommendations.

## Methods

This systematic review and meta-analysis was conducted in accordance with the WHO guideline development process[22] and the methods of the Cochrane Collaboration.[23] As part of the evidence review, results of the meta-analysis were evaluated using the methodology of the Grading of Recommendations Assessment, Development and Evaluation (GRADE) working group.[24] Evidence summaries and GRADE assessments were discussed and reviewed by the WHO Nutrition Guidance Expert Advisory Group (NUGAG) Subgroup on Diet and Health as part of WHO’s guideline development process.

The PICO questions (S1 Table) and priority health outcomes guiding this review were discussed and developed by the NUGAG Subgroup on Diet and Health. A PRISMA checklist was been completed and adhered to (S2 Table).

### Search strategy

Electronic searches were conducted in the following databases without limits on date of publication or publication language: EMBASE (to July 2016); PubMed (to July 2016); Cochrane Central Register of Controlled Trials (to July 2016); LILACS (to July 2016); and WHO Clinical Trial Registry (to July 2016). Search terms can be found in S3 Table. Reference lists of identified papers were hand-searched for additional, relevant articles.

### Inclusion criteria

Subjects included male and female children, adolescents and young adults between the ages of 2 to 19 years, from the general population. Studies considered for inclusion were those conducted in healthy individuals as well as in individuals with, or at risk of, hyperlipidaemia (including familial hypercholesterolaemia), hypertension or diabetes (type 1 or 2), or who were overweight or obese. Studies targeting those who were pregnant, acutely ill or with chronic infections such as human immunodeficiency virus (HIV) were excluded.

Randomised controlled trials (RCTs) involving interventions of at least two weeks duration with the primary intention of reducing SFA or TFA intake directly or through reduction in total fat intake, and that reported an outcome of interest were included. Trials where weight loss was the primary outcome were excluded, as were trials involving multifactorial interventions where the effect of SFA or TFA reduction could not be separated from the effect of other changes such as physical activity level. Prospective cohort studies meeting the inclusion criteria and reporting associations between SFA or TFA intake and an outcome of interest were also identified.

Outcomes of interest included total cholesterol, LDL cholesterol, high-density lipoprotein (HDL) cholesterol, triglyceride, and associated blood lipid ratios; height; body weight, body mass index (BMI) and other measures of adiposity; systolic blood pressure; diastolic blood pressure; insulin resistance and incidence of impaired glucose tolerance, impaired fasting glycaemia or type 2 diabetes; and potential adverse effects.

### Study selection

One reviewer (JM) performed the initial screen of all articles identified by the electronic searches. Articles were excluded when the title clearly indicated that it did not meet the inclusion criteria (e.g. non-human studies). Two reviewers (JM, LTM) independently reviewed the abstract and/or full text of the remaining citations. Differences in opinion as to whether particular studies should be included/excluded were resolved by discussion.

### Data extraction and quality assessment

Two reviewers (JM, LTM) independently extracted data from RCTs using standard data extraction forms, with any discrepancies resolved by discussion. Where necessary, data was extracted directly from graphs using PlotDigitizer Version 2.6.3. Cochrane criteria were used to assess risk of bias for each trial including sequence generation, allocation concealment, blinding of participants, personnel and outcome assessors, incomplete outcome data, selective outcome reporting and other possible sources of bias, which included potential differences in the groups at baseline, evidence of treatment compliance, residual confounding and systematic differences in care between control and intervention groups.[23]

### Data synthesis and statistical analysis

Meta-analysis of trial data was undertaken using the Cochrane Collaboration’s Rev Man 5.1 software [25] with generic inverse models and random-effects to account for heterogeneity resulting from differences in trial design, follow-up periods and age of participants. Effects were reported as mean differences for all outcomes except for height and body weight, which were reported as standardized mean differences to account for differences in growth rates through childhood. For trials reporting multiple follow-ups over time, the most recently published or most reliable data was used in the meta-analyses. When cross-over (paired data) studies did not report the mean difference between treatments and its standard error (SE) or other relevant statistics, end of treatment values were analysed as independent samples (a conservative approach). Effect estimates and their standard errors from correctly analysed cluster-randomized trials were included without adjustment in the meta-analyses. For incorrectly analysed cluster ranodmised trials the standard error was inflated to account for unit of analysis errors. The standard error of the effect estimate (ignoring clustering) was mulitplied by the square root of the design effect obtained from a reliable external source.[23]

Heterogeneity was assessed by visual inspection of the forest plots, and consideration of the *I*^2^ statistic, where an *I*^2^ statistic of more than 50% indicates a considerable level of inconsistency. Potential causes of heterogeneity were determined by examining characteristics of individual trials and subgroups. When more than one trial was available subgroup analyses were conducted for each outcome by the following pre-specified factors: serum lipid status; age of participants; type of intervention; trial design; duration of intervention; level of SFA or TFA reduction achieved; and the primary nutrient source replacing SFA or TFA.

Sensitivity analyses examined the effects of removing trials at high risk of bias from the analysis. A trial was considered to be at high risk of bias overall if it was graded as inadequate (high) in both the randomisation and at least one other area of bias assessment. Other trials were considered to be at low risk of bias.

Publication bias was not assessed as the number of trials found was not sufficient to conduct funnel plot analyses for any outcome measure.

## Results

### Trans-fatty acids

No trials were identified meeting the inclusion criteria. The flow of records through screening, exclusion and inclusion of trials is shown in S1 Fig.

### Saturated fatty acids

**Fig 1:**
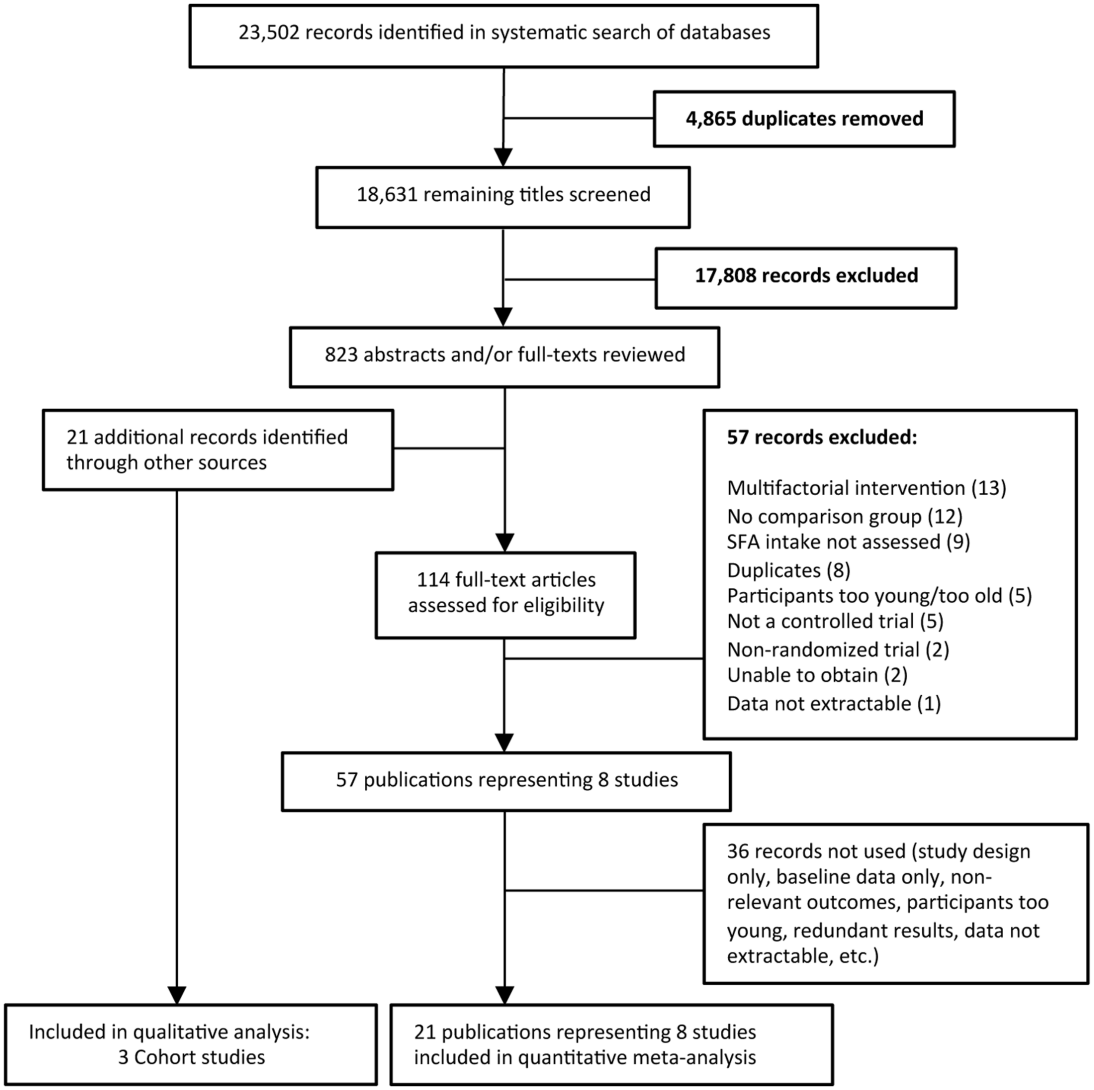
PRISMA flow diagram of trial selection for saturated fatty acid intake in children.

Electronic searches identified 23,502 potentially relevant article titles. After deduplication 18,631 articles remained. After removing citations for which a determination of eligibility could be made based on title alone, the titles, abstracts and/or full texts of 824 articles, plus an additional 21 citations identified by hand-searching were reviewed. The full texts of 114 articles were assessed for eligibility of which 57 were excluded, leaving 57 publications that represented a total of eight independent trials. After removing citations that were redundant or did not report on priority outcomes, eight independent trials reported in 21 separate articles were included in the quantitative analysis (Fig 1). The predominant reasons for exclusion were use of multifactorial interventions, not including an appropriate control group, not assessing SFA intake, and age of participants. The full text of two articles could not be obtained.[26, 27]

Trial characteristics are described in Table 1. Data from 2,430 individual children and adolescents between 2 to 19 years of age were included in the analysis. Three trials were undertaken in children and adolescents with elevated cholesterol[28–35], two in children with mixed lipid status[36–38] and three in children and adolescents for which individual cholesterol status was not reported.[39–49] Five trials had a duration of <1 year[35, 36, 38–40] and three had a duration of ≥ 1year [28–34, 41–49], including one trial with approximately 19 years of follow-up.[41–49] Four trials were conducted in the US [28–33, 37–39] and one in each of the following countries: Australia[40], China[35], Finland [41–49] and Spain.[36] One study was implemented in a school setting.[37, 38] Six trials involved parallel arm design[28–33, 35, 37, 38, 40–49], of which two were cluster randomised.[37, 38, 40] Two trials had a crossover design.[36, 39] Four trials reported assessments at multiple follow-up timepoints.[28–33, 40–49] Further detail on multi-timepoint trials can be found in S4 Table. Characteristics of the three cohort studies identified [50–52] are described in S5 Table.

**Table 1:** Characteristics of included trials.

*Study ID*	Citations	Participant characteristics			Trial characteristics						
		Lipid status	Age (years)	Number of participants	Setting	Study design	Duration (weeks)	Intervention	Exposure assessment method	Priority outcomes measured	Notes
Children’s Health Project	[32–34]	Hyperlipidemic	4–8	261 (50.2% male)	US	RCT	12 (+follow ups at 24, 52 weeks)	Dietary advice	24hr dietary recalls	LDL-C, height *z* score, weight *z* score	Two intervention and two control groups (one at-risk, one not at-risk). Not-at-risk control group not used in analysis.
Denke 2000	[39]	Not specified	8–16; mean 12	134 (53% male)	US	Crossover (cluster)	5	Dietary advice + food supplementation	Product inventory; daily consumption checklists; 3-day diet records	Total cholesterol, LDL-C, HDL-C, triglycerides, weight	
DISC	[28–31]	Hyperlipidemic	8–10	623 (54.6% male)	US	RCT	3 years (+follow-ups at 5, 7 years)	Dietary advice	24hr dietary recalls	Total cholesterol, LDL-C, HDL-C, triglycerides, height, weight, BMI, SBP, DBP	
Estevez-Gonzalez 1998	[36]	Mixed (53.4% hyperlipidemic)	3–9	88 (54.5% male)	Spain	Crossover	28	Food supplementation	FFQ	Total cholesterol, LDL-C, HDL-C, triglycerides, ApoA1, ApoB	
Healthy Start	[37, 38]	Mixed (37.8% hyperlipidemic)	2–5; mean 4.4	585 (51% male)	US	RCT (cluster)	36	Dietary advice + food supplementation	24hr dietary recalls	Total cholesterol	Two intervention groups: one diet modification only, one diet modification plus nutrition counselling. Both intervention groups combined for analysis.
Hendrie 2011	[40]	Not specified	3–9	137 (60% male)	Australia	RCT (cluster)	24	Dietary advice	24hr dietary recalls; serum pentadecanoic acid levels	Total cholesterol, LDL-C, HDL-C, triglycerides, height*, weight*, BMI*, BMI *z* score, waist circumference	Used follow-up effect estimates in the meta-analysis assuming that these have been adjusted for cluster randomization; height, weight and BMI were not adjusted for clustering and therefore not included in meta-analysis
STRIP	[41–49]	Not specified (randomized at 7 months of age)	3 (first report relevant to this review)	442 (50% male) at 19–20 years of age	Finland	RCT	19 years of follow up	Dietary advice	3-day diet records	Total cholesterol, LDL-C, HDL-C, nonHDL-C, triglycerides, ApoA1, ApoB height, relative height, weight, relative weight, BMI, SBP, DBP, HOMA IR, waist circumference	Participants received intervention beginning at 7 months of age (delivered to parents initially)
Zhu 2003	[35]	Hyperlipidemic	7–11	160 (44% male)	China	RCT	12	Dietary advice	3-day diet records	Total cholesterol, LDL-C, HDL-C, triglycerides, ApoA1, ApoB	Data on height, weight, waist circumference, SBP and DBP was not reported in sufficient detail to include in the meta analysis.

LDL-C, LDL cholesterol; HDL-C, HDL cholesterol; BMI, body mass index; SBP, systolic blood pressure; DBP, diastolic blood pressure; ApoA1, apolipoprotein A1; ApoB, apolipoprotein B; HOMA IR, homeostasis model of insulin resistance

#### Interventions

In five trials the intervention consisted solely of providing dietary advice or counselling designed to reduce SFA intake.[28–32, 35, 40, 45] In one trial the intervention group was provided with foods where fat quality was modified[36] and in another both dietary advice and fat-modified foods were provided to the intervention group.[39] One trial had two intervention arms, one providing modified foods and one providing both modified food and dietary advice.[37, 38] In trials that provided food, two gave supplemental foods and spreads to participants to be consumed at home[36, 39] and one provided food through modified cafeteria meals delivered in a pre-school setting (approximately one third of their daily calories).[37, 38] All five trials providing dietary advice incorporated counselling sessions with dietitians, paediatricians and other qualified staff, however one of these included two intervention groups where one group received counselling and the other a home-based, self-led education intervention without counselling.[32, 33]

SFA intake was assessed using 24hr dietary recalls in four trials[28–33, 37, 38, 40], 3-day diet records in three trials[35, 39, 41–49], and food frequency questionnaires and/or checklists in two trials.[36, 39] One study also used biomarker assessment (pentadecanoic acid).[40]

#### Blood lipids

**Fig 2:**
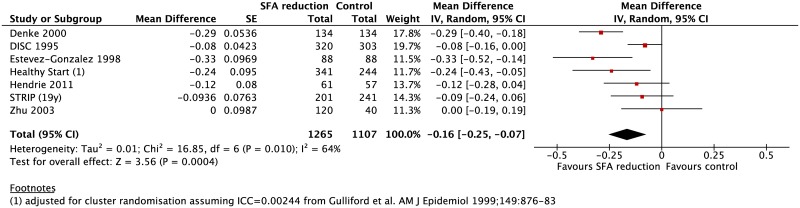
Meta-analysis of weighted mean (95% CI) differences in effects on total cholesterol (mmol/l) in randomised trials that compared usual diets with reduced saturated fat diets in children.

**Fig 3:**
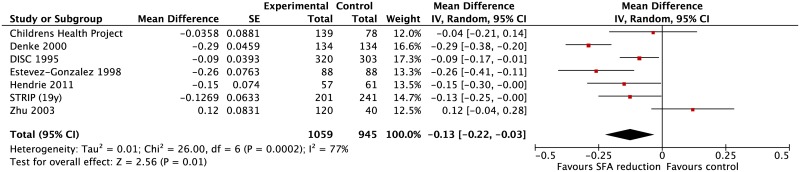
Meta-analysis of weighted mean (95% CI) differences in effects on LDL cholesterol (mmol/l) in randomised trials that compared usual diets with reduced saturated fat diets in children.

Seven trials reported effects on total cholesterol[28, 35–40, 48] and LDL-cholesterol. [28, 33, 36, 39, 40, 48] Compared with control diets, there was a highly statistically significant effect of reduced SFA intake on total cholesterol (mean difference (MD) -0.16 mmol/l [95% confidence interval (CI): -0.25 to -0.07]) (Fig 2) and LDL cholesterol (MD -0.13 mmol/l [95% CI: -0.22 to -0.03]) (Fig 3). Moderate to significant heterogeneity was observed for both total cholesterol (I^2^ = 64%) and LDL cholesterol (I^2^ = 77%). This could be explained entirely by differences in trial design and/or the nature of the nutrient replacing SFA; subgroup analysis revealed statistically significantly greater reductions in total cholesterol (MD -0.30 mmol/L [95%CI: -0.39, -0.21]) and LDL cholesterol (MD -0.28 mmol/l [95%CI: -0.36, -0.20]) in two crossover trials.[36, 39] These trials exchanged SFA with PUFA-rich unsaturated fatty acids (controlled through provision of fat-containing foods) and achieved greater differences in SFA intake between control and intervention children than in the parallel design trials (Table 2). In contrast the reductions in total cholesterol (MD -0.10 mmol/l [95%CI: -0.15 to -0.04]) and LDL cholesterol (MD -0.07 mmol/l [95%CI: -0.15 to 0.01]) were smaller in parallel trials where more general advice was given regarding replacement of SFA (P = 0.0002 for the subgroup differences for both comparisons) (S2 and S3 Figs).

**Table 2:** Dietary intake data for included trials.

Study ID	Achieved SFA intake	Replacement nutrient^a^
Children’s Health Project	Int 1: 10.5% EI^b^ Int 2: 10.2% EI Cont: 11.6% EI	Not specified
Denke 2000	Int: 9% EI^c^ Cont: 16% EI	PUFA (3→10% EI), TFA (0.5→1.5% EI)
DISC	Int: 10.2% EI^c^ Cont: 12.3% EI	Protein (14.8→16.0% EI), carbohydrate (53→56.2% EI)
Estevez-Gonzalez 1998	Not reported^d^	MUFA, PUFA^e^
Healthy Start	Int: 11.6% EI Cont: 12.4% EI	Partially by protein (incomplete dietary intake data)
Hendrie 2011	Int: 13.3% EI^c^ Cont: 16.6% EI	Protein (16.1→17.0%), carbohydrate (48.2→49.6%), PUFA (4.0→4.2)
STRIP (at 19y of age)	Int: 11.6% EI (males:11.8%; females: 11.4%)^c^ Cont: 12.3% EI (males:11.4%; females: 12.0%)^f^	Partially by protein and carbohydrate (incomplete dietary intake data at 19 year follow-up)
Zhu 2003	Int: 7.7% EI Cont: 2.7% EI	Carbohydrate intake increased in intervention group (38.4→51.8% EI)

Int, intervention group; Cont, control group; %EI, % of total daily energy intake; SFA, saturated fatty acids; PUFA, polyunsaturated fatty acids; TFA, trans fatty acids; MUFA, monounsaturated fatty acids

^a^ Based on dietary intake data

^b^ As assessed at three month follow-up of a one year trial (data not sufficient to calculate for later timepoints).

^c^ p < 0.05

^d^ The fat content of the food supplements provided 26.2% of total fat intake to the participants, 3.9% of which was saturated fatty acids in the intervention group and 17.4% of which was saturated fatty acids in the control group. Total SFA intake was not reported, however, estimates based on other dietary information provided about the trial indicate that the whole milk (control) provided approximately 6.5% of total energy intake as SFA and the milk preparation (interventions) provided approximately 1.4% of total energy intake as SFA. As the milk/milk preparation provided 26.2% of total fat intake, it is very likely that those consuming whole milk were consuming >10% of total energy intake as SFA. It is less clear if those consuming the milk preparation achieved an SFA intake of <10% of total energy intake.

^e^ Food supplement provided to intervention group contained 70% MUFA, 15% PUFA, 15% SFA. Food supplement provided to control group contained 30% MUFA, 3% PUFA, 67% SFA.

^f^ Actual SFA intake varied throughout 19 years of follow-up but was consistently lower in intervention groups

Subgroup analysis of the effect of the initial lipid status of the participants found a significantly different effect of SFA reduction on total cholesterol (*p* = 0.05) between trials involving normolipidaemic/mixed status children (MD -0.21 mmol/l [95% CI: -0.31 to -0.12]) *vs* hyperlipidaemic children (MD -0.08 mmol/l [95% CI: -0.15 to -0.01]) (S4 Fig). Similarly the reduction in LDL cholesterol was greater among the trials conducted in normolipidaemic/mixed status (MD -0.21 mmol/l [95% CI: -0.30 to -0.13]) *vs* hyperlipidaemic children (MD -0.02 mmol/l [95% CI: -0.14 to -0.11]) (S5 Fig).

Two trials reported achieving SFA intakes of less than 10% of total energy intake (the current WHO population nutrient intake goal[53]) in the intervention group.[35, 39] One of these had a high risk of bias, presented data that did not adequately account for baseline differences in lipid levels and reported null effects.[35] The other was a well-designed cluster randomised crossover trial in which the reductions in total (MD -0.29 mmol/l [95% CI: -0.40 to -0.18]) and LDL cholesterol (MD -0.29 mmol/l [95% CI: -0.38 to -0.20]) were significantly greater than the pooled effects for the five trials where SFA intake was greater than 10% of total energy intake in the intervention group (*p* = 0.03 and *p* = 0.003 for subgroup differences, respectively)[39] (S6 and S7 Figs).

There were no significant associations observed for HDL cholesterol, triglycerides or apolipoproteins A1 or B (Table 3).

**Table 3:** Summary of effect estimates for randomised trials that compared usual diets with reduced saturated fat diets in children.

Outcome	Trials	Participants^a^ (n)	Effect Estimate (95%CI)	*p* value	I^2^
Total cholesterol (mmol/L)	7	2150	-0.16 [-0.25, -0.07]	0.0005	64%
LDL cholesterol (mmol/L)	7	1782	-0.13 [-0.22, -0.03]	0.01	77%
HDL cholesterol (mmol/L)	6	1565	0.00 [-0.02, 0.02]	0.82	23%
Triglycerides (mmol/L)	6	1565	-0.02 [-0.06, 0.01]	0.22	20%
BMI (kg/m^2^)	3	1189	-0.10 [-0.32, 0.12]	0.36	0%
Body weight (SMD)	4	1419	-0.03 [-0.13, 0.07]	0.55	0%
Height (SMD)	3	1287	0.09 [-0.03, 0.21]	0.16	11%
Waist circumference (cm)	2	576	-0.20 [-1.38, 0.98]	0.28	0%
Systolic blood pressure (mm Hg)	2	1106	-0.68 [-1.71, 0.35]	0.19	0%
Diastolic blood pressure (mm Hg)	2	1106	-1.45 [-2.34, -0.56]	0.001	0%
Apolipoprotein A1 (mg/dL)	3	778	-1.03 [-3.95, 1.90]	0.69	7%
Apolipoprotein B (mg/dL)	3	778	-1.25 [-6.26, 3.76]	0.62	70%
Insulin resistance (HOMA-IR)	1	437	-0.14 [-0.28, 0.01]	0.06	NA

^a^ subjects in crossover trials counted once only

#### Anthropometric measures

There were no significant associations observed between reduced SFA intake and weight, height, body mass index (BMI) or waist circumference (Table 3).

#### Blood pressure

**Fig 4:**
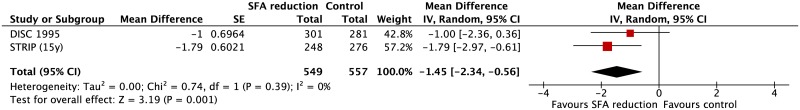
Meta-analysis of weighted mean (95% CI) differences in effects on diastolic blood pressure (mm Hg) in randomised trials that compared usual diets with reduced saturated fat diets in children.

**Fig 5:**
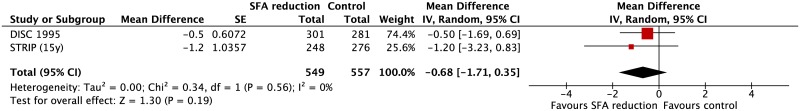
Meta-analysis of weighted mean (95% CI) differences in effects on systolic blood pressure (mm Hg) in randomised trials that compared usual diets with reduced saturated fat diets in children.

Three trials reported blood pressure outcomes [29, 35, 47] but only two reported data in sufficient detail for the meta-analysis. [29, 47] A statistically significant decrease in diastolic blood pressure associated with reduced SFA intake was observed (MD -1.45 mm Hg [95% CI: -2.34 to -0.56]) (Fig 4). There were no significant effects of SFA reduction on systolic blood pressure (Fig 5). In addition, one of these trials separately reported on risk of high blood pressure and found that reduced SFA intake reduced the risk of high blood pressure in adolescents (relative risk [RR] 0.83 [95% CI: 0.70 to 0.99]).[54]

#### Insulin sensitivity

One trial reported improvements in insulin sensitivity as measured by the homeostasis model of insulin resistance (HOMA-IR) at 9 years of age[46] (*p* = 0.02 for intervention effect) and between 15–20 years[49] (*p*_trend_ = 0.005 for intervention effect). At age 19 the MD was -0.14 [95%CI: -0.28, 0.01] (Table 3). Multivariate analyses suggest that the improvements were largely due to reduced SFA saturated fat intakes, but increased dietary fibre intakes may have also have been a factor.

#### Adverse effects

In addition to no observed adverse effects on anthropometric measures, there was no evidence of adverse effects of reducing SFA intake in children on micronutrient intakes, cognitive development or sexual maturation in the small number of trials reporting these outcomes.

Two trials assessed micronutrient intakes.[28, 31, 42] One noted that self-reported intakes of some micronutrients were lower in the reduced SFA group, however no adverse effects were observed in serum indicators of nutritional status and there was no significant difference in adjusted serum ferritin levels (MD -2.1 μg/L [95%CI: -4.9 to 0.8]; *p* = 0.08) between groups. This effect was reduced to 1.5 μg/L (*p* = 0.12) when an outlier was removed. [28, 31] Similarly, serum ferritin levels in 3–4 year old children participating in a second trial were not different between groups (MD -2.6 μg/L [95%CI: -8.2 to 2.9]; *p* > 0.05), nor were there differences observed in intakes or other indicators of iron and zinc status.[42]

Two trials assessed the effect of SFA reduction on cognitive development. There were no significant differences in the relative risk of failing tests of speech and language skills, gross motor functioning plus perception, or in visual motor skills for 5 year old children in the intervention compared with the control group.[44] In addition, there was no difference in parental perceptions of their children’s behavior at 3 years between the intervention and control groups.[43] Similarly, children in another trial exhibited no signs of adverse effects of SFA reduction in terms of academic functioning, psychological symptoms or family functioning.[30]

In one trial that assessed sexual maturation, reduced saturated fat intake had no effect on Tanner stages of puberty in males or females at any point during the trial.[31, 55, 56]

#### Cohort studies

Three prospective cohort studies were identified that reported on associations between SFA intake at baseline, or changes in SFA intake during follow-up, and relevant health outcomes in children. The Bogalusa Heart Study compared total and LDL cholesterol concentrations in children who had maintained consistent SFA intakes during 6.5 years of follow-up.[57] Total and LDL cholesterol concentrations of the children in the highest *vs* lowest tertiles of SFA intake were compared but no significant association was found. Magarey et al. examined the association between SFA intake (measured periodically over the 15 years) and measures of adiposity, and found a statistically significant association between energy adjusted SFA intake and BMI standard deviation score after adjustment for baseline fatness, sex and parental BMI.[52] The Avon Longitudinal Study of Pregnancy and Childhood reported significant associations from reverse stepwise multiple regression models examining the effect of nutrient intakes (assessed at 18 months of age) on cholesterol concentrations at 31 months of age.[50] There was a significant association between SFA intake and total cholesterol in boys and on the total:HDL cholesterol ratio in girls. Data from the cohort studies was not included in the meta-analysis or GRADE assessment.

### Quality of the body of evidence

**Fig 6:**
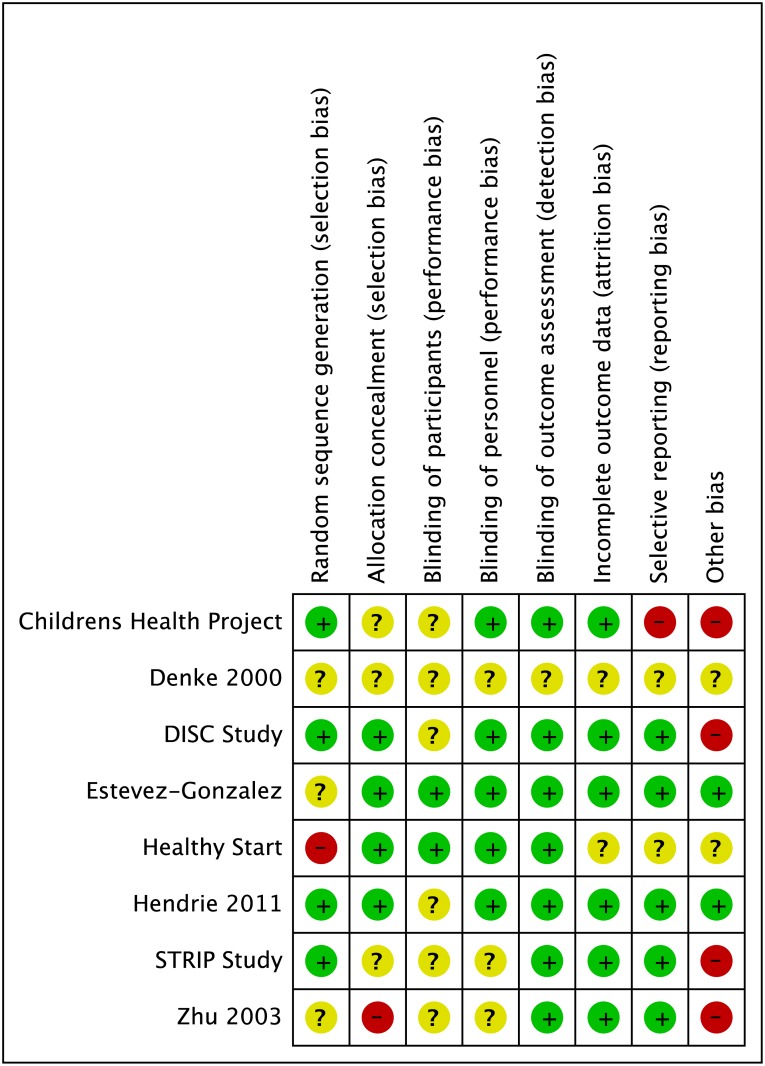
Risk of bias assessment for included trials + = low risk of bias; ? = unclear risk of bias; - = high risk of bias.

Some degree of bias was detected for most trials (Fig 6) but only one trial was assessed as being at high risk of bias over a range of criteria. Three trials had a high risk of bias for possible differences in care between intervention and control group[28–33, 41–49], one of which also had a high risk of bias for selective reporting.[32, 33] One trial had high risk of bias in terms of randomisation[37, 38]. Another raised further questions relating to allocation concealment and other areas of potential bias, suggesting high risk of bias overall[35]. Sensitivity analyses examining the influence of this trial showed that its exclusion slightly strengthened the associations between SFA reduction and total and LDL cholesterol but did not change our interpretation of the overall findings.

Assessment of the quality of the results for outcomes included in the main analysis can be found in GRADE evidence profile 1 (S6 Table). All outcomes for children were rated as “high” with the exception of triglyceride, apolipoprotein A1, systolic blood pressure and insulin resistance which were rated as “moderate”. Similar assessments were made for threshold and replacement analyses (GRADE evidence profiles 2–5, S6 Table).

## Discussion

This systematic review and meta-analysis showed that reduction of SFA intake in children and adolescents between 2 to 19 years of age was associated with statistically significant reductions in total and LDL cholesterol and diastolic blood pressure. These effects were consistent across all of the trials included in the meta-analyses. The effects on cholesterol were greatest among those in which SFA was replaced primarily with PUFA or MUFA, and when the intervention group achieved a reduction in SFA to below 10% of total energy intake.

### Findings in Context

The very low incidence of cardiovascular events in children makes an examination of the direct effects of saturated fat intake on CVD impractical. Total and LDL cholesterol can readily be measured in children, however, and both are well established risk factors for coronary heart disease in adults; a meta-analysis of prospective cohort studies involving over 900,000 adults found a linear association between blood cholesterol concentrations and coronary mortality[6], while clinical trials show that cholesterol- lowering with statin therapy reduces coronary heart disease risk.[51] Furthermore, elevated LDL cholesterol in children has been found to be associated with preclinical signs of atherosclerosis.[12]

There is strong evidence from controlled clinical trials in adults that replacing dietary SFA with MUFA, PUFA or carbohydrate reduces total and LDL cholesterol concentrations.[1, 3] In addition there is reasonably strong evidence from randomised trials in adults that replacing SFA with PUFA from plant oils, but not refined carbohydrates, reduces the incidence of cardiovascular events and mortality. [8–10] However recent meta-analyses of randomised trials examining the effect of replacing saturated fat with plant oils rich in linoleic acid (n-6 PUFA), including recovered data from Sydney Diet Heart Study (SDHS) and the Minnesota Coronary Survey (MCS), both conducted over 50 years ago, indicates no benefit with respect to coronary heart disease mortality despite reduction in total cholesterol. [18, 19] These new findings should be viewed with caution given that in the SDHS and MCS intakes of PUFA intervention groups exceeded current recommendations, very high rates of loss to follow-up occurred, there was evidence of selective outcome reporting and neither study was powered to show an effect of n-6 PUFA on mortality.[20, 21] We believe our findings in children are compatible with the totality of evidence in adults indicating beneficial effects in terms of lower coronary heart disease risks when replacing dietary SFA with PUFA-rich unsaturated fatty acids.[36, 39]

Questions have been raised about the appropriateness of recommending low-fat diets to children over concerns that excessive restriction of dietary fat could result in inadequate nutrient intakes—particularly of iron—which could negatively impact normal growth and development.[58, 59] In the analysis there was no evidence of adverse effects of reducing SFA intake on anthropometric measures of growth, cognitive development or micronutrient intake, including iron. A primary focus of both the Dietary Intervention Study in Children (DISC)[28] and Special Turku Coronary Risk Factor Intervention Project (STRIP)[41] was to assess the safety of a reduced SFA diet in children in terms of growth and development, and authors of both trials concluded that a diet low in SFA was safe for children.

That no relevant trials of TFA intake in children were found may be because most epidemiological studies on TFA intake have assessed cardiovascular outcomes which generally only present in later life. Several trials assessing the effects of conjugated linoleic acid (CLA) supplementation on weight loss in children were identified but none met the inclusion criteria.[58, 60–63]

### Strengths and limitations

A strength of this review is the quality and consistency of the data, particularly that relating to total and LDL cholesterol. Results of the meta-analysis are also highly consistent with what has been observed in trials in adults. The data covers children between 2 to 19 years of age and consistently showed that advice given to parents and to older children was effective in reducing SFA intakes. Data from the STRIP trial[41–49] reporting effects across the course of childhood through to adulthood illustrates this consistency (S8 Fig). Adolescents in the DISC trial similarly demonstrated seemingly consistent levels of compliance over three years of follow-up[28–31].

These findings are subject to the limitations inherent to most dietary intervention studies, notably difficulties in obtaining reliable dietary intake data, maintaining the blind among participants and personnel, and variation in the nature and quality of the interventions, all of which may bias the results. It is generally accepted that it becomes increasingly difficult for participants to maintain adherence to dietary interventions over time although this may be more of an issue in trials involving adults with previously established eating habits. Since children are typically reliant on adults to provide nourishment, they may be less prone to stray from the intervention diet, provided caregivers comply with the intervention. The possibility that the effects may be partially explained by factors other than saturated fat intakes such as differences in consumption of fruit and vegetables or physical activity levels between control and intervention groups cannot be ruled out. Although the primary objective of the STRIP trial was fat modification[41], some reports of follow-up conducted later in later childhood and adolescence indicate that the intervention group received additional advice regarding intake of fruit, vegetables and dietary fibre, smoking and physical activity.[54] Thus we cannot be entirely certain the effects observed were primarily due to SFA reduction. However, the significant effects on total and LDL cholesterol remain even when the STRIP data is excluded from the analyses (data not shown). Given the careful consideration of the potential sources of bias described above in assessing the quality of the evidence, and the observation that the greatest effects on cholesterol levels were seen in the most controlled trials, our confidence in the results of the meta-analysis is high.

## Conclusion

Advice to reduce saturated fat intake of children results in a significant reduction in total and LDL-cholesterol levels as well as diastolic blood pressure without adverse effects. Results of this review suggest that the greatest effect on cholesterol occurred when SFA was replaced with PUFA or a mixture of PUFA/MUFA, although benefit was observed even when replacement was unclear. Results further suggest that the greatest reduction in cholesterol occurred when SFA intake was less than 10% of total energy intake. Elevated cholesterol has been linked to CVD in adults and preclinical markers of atherosclerosis in children, thus reducing saturated fat intake from an early age may help to reduce the risk of CVD later in life. CVD is a major contributor to the substantial global economic burden of NCDs. Projected increases in NCDs over the next two decades could cost up to 75% of global gross domestic product (GDP) as a result of healthcare costs and lost productivity due to death or disability.[64] Interventions targeting reduction in saturated fat intakes amongst children and adolescents could translate into major cost savings by reducing risk of CVD. Interventions should focus on reducing intakes of highly processed fried and nutrient-poor fast foods and snacks, processed meats and fatty meats. High-fibre fruits and vegetables, nuts and seeds, lean meats and reduced fat dairy foods should be the core components of children’s diets.

## Supporting information

S1 Table Questions that the review attempted to address. (DOCX)

S2 Table PRISMA 2009 checklist. (PDF)

S3 Table Search terms used in the literature review for saturated and trans fatty acids. (DOCX)

S4 Table Notes on multi-timepoint trials. (DOCX)

S5 Table Characteristics of cohort studies. (DOCX)

S6 Table Grade evidence profiles 1–6. (DOCX)

S1 Fig PRISMA flow diagram of trial selection for TFA intake in children. (TIFF)

S2 Fig Effect of saturated fat intake on total cholesterol (mmol/l): Subgroup analysis by trial design. (EPS)

S3 Fig Effect of saturated fat intake on LDL cholesterol (mmol/l): Subgroup analysis by trial design. (EPS)

S4 Fig Effect of saturated fat intake on total cholesterol (mmol/l): Subgroup analysis by initial lipid status of trial participants. (EPS)

S5 Fig Effect of saturated fat intake on LDL cholesterol (mmol/l): Subgroup analysis by initial lipid status of trial participants. (EPS)

S6 Fig Effect of saturated fat intake on total cholesterol (mmol/l): Subgroup analysis by level of SFA intake. (EPS)

S7 Fig Effect of saturated fat intake on LDL cholesterol (mmol/l): Subgroup analysis by level of SFA intake. (EPS)

S8 Fig Effect of saturated fat intake on total cholesterol (mmol/l); Special Turku Coronary Risk Factor Intervention Project (STRIP) reports. (TIFF)
