# Supplementary material for: Health effects of saturated and trans-fatty acid intake in children and adolescents: Systematic review and meta-analysis
Source: PLoS One. 2017 Nov 17;12(11):e0186672. doi: 10.1371/journal.pone.0186672 (PMC5693282; doi:10.1371/journal.pone.0186672)
Supplement: S1 Table — (DOCX) [file pone.0186672.s001.docx]

**S Table 1**. Questions that the review attempted to address

|  | **PICO questions** |
| --- | --- |
| **Saturated fatty acids** | 1. What is the effect in the population of reduced percentage of total energy (%E) intake from saturated fatty acids (SFA) relative to higher intake for reduction in risk of noncommunicable diseases (NCDs)?  2. What is the effect in the population of consuming <10%E as SFA relative to >10%E as SFA for reduction in risk of NCDs?  3. What is the effect in the population of a reduction in %E from SFA from 10% in gradual increments relative to higher intake for reduction in risk of NCDs?  4. What is the effect in the population of reduced %E intake from long-chain SFA, very long-chain SFA and medium-chain SFA relative to higher intake for reduction in risk of NCDs?  5. What is the effect in the population of reduced %E intake from lauric acid/ myristic acid/ palmitic acid/ stearic acid relative to higher intake for reduction in risk of NCDs?  6. What is the effect in the population of replacing SFA with polyunsaturated fatty acids (PUFAs)/ monounsaturated fatty acids (MUFAs)/ carbohydrates (refined vs. unrefined)/ protein/ trans fatty acids (TFAs) relative to no replacement for reduction in risk of NCDs? |
| **Trans fatty acids** | 1. What is the effect in the population of reduced percentage of total energy (%E) intake from trans- fatty acids (TFA) relative to higher intake for reduction in risk of non-communicable diseases (NCDs)?  2. What is the effect in the population of a reduction in %E from TFA from 1% in gradual increments relative to higher intake for reduction in risk of NCDs?  3. What is the effect in the population of reduced %E from industrial/ruminant TFA relative to higher intake for reduction in risk of NCDs?  4. What is the effect in the population of consuming 0%E industrial/ruminant TFA relative to >0%E industrial/ ruminant TFA intake for reduction in risk of NCDs?  5. What is the effect in the population of reduced %E from 18:2n-6/ 18:3n-3 isomers of TFA relative to higher intake for reduction in risk of NCDs?  6. What is the effect in the population of replacing %E from TFA with conjugated linoleic acid (CLA) isomers [9-cis, 11-trans conjugated linoleic acids (CLAs) and 10-trans, 12-cis CLAs]?  7. What is the effect in the population of replacing TFA with saturated fatty acids (SFA)/ polyunsaturated fatty acids (PUFA)/ monounsaturated fatty acids (MUFA)/ carbohydrate (refined vs. unrefined) relative to no replacement or reduction in risk of NCDs? |
