# Supplementary material for: Health effects of saturated and trans-fatty acid intake in children and adolescents: Systematic review and meta-analysis
Source: PLoS One. 2017 Nov 17;12(11):e0186672. doi: 10.1371/journal.pone.0186672 (PMC5693282; doi:10.1371/journal.pone.0186672)
Supplement: S3 Table — (DOCX) [file pone.0186672.s003.docx]

**S2 Table**.

**Search terms used in the literature review for saturated fatty acids**

**P**

**ubMed**

#1 (“Fatty Acids” [MeSH Terms:noexp] OR “Myristic Acids” [MeSH] OR “Palmitic Acids” [MeSH] OR “Stearic Acids” [MeSH] OR "Lauric Acids" [MeSH] OR "Dietary Fats/administration and dosage" [MeSH] OR “Dietary Fats” [MeSH Terms:noexp] OR “Butter” [MeSH] OR Diet, Fat-Restricted [MeSH] OR saturated* [tiab] OR "myristic" [tiab] OR myristate* [tiab] OR "palmitic" [tiab] OR palmitate* [tiab] OR "stearic" [tiab] OR stearate* [tiab] OR "lauric" [tiab] OR laurate* [tiab] OR "long chain fatty acids" [tiab] OR "very-long chain fatty acids" [tiab] OR "medium chain fatty acids" [tiab] OR dairy fat* [tiab] OR dietary fat* [tiab] OR "fat intake" [tiab] OR “reduced fat” [tiab] OR "reducing fat" [tiab] OR “low fat” [tiab] OR "lower fat" [tiab] OR "lowered fat" [tiab] OR "modified fat" [tiab] OR "modifying fat" [tiab] OR animal fat* [tiab] OR “butter” [tiab] OR “coconut oil” [tiab] OR “palm oil” [tiab] OR “palm kernel oil” [tiab] OR “peanut oil” [tiab])

#2 ("cholesterol" [MeSH] OR "lipoproteins" [MeSH] OR blood lipid* [tiab] OR “cholesterol" [tiab] OR "Hypercholesterolemia"[Mesh] OR lipoprotein* [tiab] OR “LDL” [tiab] OR “HDL” [tiab] OR “LDL/HDL” [tiab] OR “total/HDL” [tiab] OR “apolipoprotein(a)” [tiab] OR “apo(a)” [tiab])

#3 ("triglycerides" [MeSH] OR "Hypertriglyceridemia"[Mesh] OR triglycer* [tiab] OR triacylgly* [tiab] OR “TG/HDL” [tiab] OR hypertriglycer* [tiab])

#4 (("body weight" [MeSH] NOT (“Birth weight” [MeSH] OR “Fetal weight” [MeSH] OR “Thinness” [MeSH])) OR "body mass index" [MeSH] OR "waist circumference" [MeSH] OR "obesity" [MeSH] OR "waist-hip ratio" [MeSH] OR "body fat distribution" [MeSH] OR “Skinfold thickness” [MeSH] OR "Abdominal Fat/drug effects"[Mesh] OR "Abdominal Fat/growth and development" [Mesh] OR “body mass” [tiab] OR "weight” [tiab] OR "weights” [tiab] OR "BMI" [tiab] OR "overweight" OR "obesity" [tiab] OR “obese” OR "waist circumference" [tiab] OR "waist circumferences" OR "waist-hip" [tiab] OR "waist-to-hip" [tiab] OR "body fat" [tiab] OR "body fats" [tiab] OR adipos* [tiab] OR “percentage body fat” [tiab] OR “metabolic syndrome”)

#5 ("blood pressure" [MeSH] OR "hypertension" [MeSH] OR blood pressure* [tiab] OR "hypertension" [tiab] OR "systolic" [tiab] OR "diastolic" [tiab])

#6 ("diabetes mellitus, type 2" [MeSH] OR “diabetes” [tiab] OR "MODY" [tiab] OR "NIDDM" [tiab])

#7 ("insulin resistance" [MeSH] OR "glucose Intolerance" [MeSH] OR "blood glucose" [MeSH] OR "hyperglycemia" [MeSH] OR "insulin" [tiab] OR "glucose" [tiab] OR hyperglycem* [tiab] OR hyperinsulin* [tiab] OR insulin sensitiv* [tiab])

#8 ("Growth and Development" [Mesh:NoExp] OR "Growth" [Mesh:NoExp] OR "Body Size" [Mesh NoExp] OR “Body height” [MeSH] OR "Adolescent Development" [Mesh] OR "Child Development" [Mesh] OR "growth" [tiab] OR "development" [tiab])

#9 ("child"[MeSH] OR "adolescent"[MeSH] OR "young adult"[MeSH] OR "child"[tiab] OR "children"[tiab] OR "adolescent"[tiab] OR "adolescents"[tiab] OR "adolescence"[tiab] OR "teen"[tiab] OR "teens"[tiab] OR "teenager"[tiab] OR "teenagers"[tiab] OR "youth"[tiab] OR "youths"[tiab] OR "childhood"[tiab] OR "young adult"[tiab] OR "young adults"[tiab])

**((#1 AND #2) OR (#1 AND #3) OR (#1 AND #4) OR (#1 AND #5) OR (#1 AND #6) OR (#1 AND #7) OR (#1 AND #8) AND #9) NOT ("animals"[MeSH] NOT "humans"[MeSH])**

**EMBASE**

| **#** | **Searches** |
| --- | --- |
| 1 | 'fatty acid'/de OR 'myristic acid derivative'/exp OR 'palmitic acid derivative'/exp OR 'stearic acid derivative'/exp OR 'lauric acid derivative'/exp OR 'fat intake'/de OR 'edible oil'/de OR 'butter'/exp OR 'low fat diet'/exp OR saturated*:ti,ab OR ‘myristic’:ab,ti OR myristate*:ti,ab OR ‘palmitic’:ab,ti OR palmitate*:ti,ab OR ‘stearic’:ab,ti OR stearate*:ti,ab OR ‘lauric’:ab,ti OR laurate*:ti,ab OR ‘long chain fatty acids’:ab,ti OR ‘very-long chain fatty acids’:ab,ti OR ‘medium chain fatty acids’:ti,ab OR ‘dairy fat’:ti,ab OR ‘dairy fats’:ti,ab OR ‘dietary fat’:ti,ab OR ‘dietary fats’:ti,ab OR ‘reduced fat’:ti,ab OR ‘reducing fat’:ti,ab OR ‘low fat’:ti,ab OR ‘modified fat’:ti,ab OR ‘modifying fat’:ti,ab OR ‘coconut oil’:ti,ab OR ‘palm oil’:ti,ab OR ‘palm kernel oil’:ti,ab OR ‘peanut oil’:ti,ab OR ‘butter’:ti,ab OR 'animal fats':ti,ab OR ‘animal fat':ti,ab |
| 3 | 'cholesterol'/exp OR ‘ lipoproteins’ OR ‘blood lipids’ OR ‘blood lipid’ OR ‘cholesterol’ OR ‘apolipoprotein’ OR ‘cholesteremia’ OR ‘ cholesterinemia’ OR ‘ cholesterolemia’ OR ‘hypercholesteremia’ OR ‘hypercholesterinaemia’ OR ‘hypercholesterinemia’ OR ‘hypercholesterolaemia’ OR ‘beta lipoprotein’ OR ‘ LDL’ OR ‘HDL’ OR ‘apo a’ OR ‘lipoprotein’ |
| 4 | 'triacylglycerol'/exp OR 'triacylglycerol' OR ‘ triacyl glyceride’ OR ‘ triglyceride’ OR ‘ triglycerides’ OR ‘ tryglyceride’ OR ‘hypertriglyceridaemia’ OR triglycer*:ti,ab OR triacylgly*:ti,ab OR ‘TG/HDL’ OR hypertriglycer*:ti,ab |
| 5 | 'body mass'/exp OR ‘BMI (body mass index)’ OR ‘body ban mass’ OR ‘body mass index’ OR ‘Quetelet index’ OR 'waist circumference'/exp OR ‘adipose tissue hyperplasia’ OR ‘adipositas’ OR ‘ diposity’ OR ‘alimentary obesity’ OR ‘ body weight, excess’ OR ‘fat overload syndrome’ OR ‘nutritional obesity’ OR ‘obesitas’ OR ‘overweight’ OR 'obesity'/exp OR ‘hip to waist ratio’ OR ‘hip waist ratio’ OR ‘waist-hip ratio’ OR ‘waist to hip ratio’ OR 'waist hip ratio'/exp OR ‘adipose tissue distribution’ OR ‘fat tissue distribution’ OR ‘fatty tissue distribution’ OR ‘subcutaneous fat distribution’ OR ‘visceral fat distribution’ OR 'body fat distribution'/exp OR ‘skin fold measurement’ OR ‘skin fold thickness’ OR ‘skin thickness’ OR ‘skinfold measurement’ OR 'skinfold thickness'/exp OR (('abdominal fat'/exp OR ‘abdominal adipose tissue’) AND 'drug effect'/exp) OR ‘body mass’:ti,ab OR ‘weight’:ti,ab OR ‘weights’:ti,ab OR ‘BMI’:ti,ab OR ‘overweight’ OR ‘obesity’:ti,ab OR “obese” OR ‘waist circumference’:ti,ab OR ‘waist circumferences’:ti,ab OR ‘waist-hip’:ti,ab OR ‘waist-to-hip’:ti,ab OR ‘body fat’:ti,ab OR ‘body fats’:ti,ab OR adipos*:ti,ab OR ‘metabolic syndrome’:ti,ab |
| 6 | 'body weight'/exp NOT ( 'birth weight'/exp OR 'fetus weight'/exp) |
| 7 | #5 OR #6 |
| 8 | 'hypertension'/exp OR 'blood pressure'/exp OR 'blood pressure monitoring'/exp OR ‘hypertensive disease’ OR ‘hypertensive effect’ OR ‘hypertensive response’ OR ‘systolic’ OR ‘diastolic’ |
| 9 | 'non insulin dependent diabetes mellitus'/exp OR MODY OR ‘adult onset diabetes’ OR ‘ adult onset diabetes mellitus’ OR ‘ diabetes mellitus type 2’ OR ‘ diabetes mellitus type ii’ OR ‘ diabetes mellitus, non-insulin-dependent’ OR ‘ diabetes mellitus, type 2’ OR ‘ diabetes mellitus, type II’ OR ‘ diabetes mellitus, maturity onset’ OR ‘ diabetes mellitus, non insulin dependent’ OR ‘ diabetes type 2’ OR ‘ diabetes type II’ OR ‘ diabetes, adult onset’ OR ‘ dm 2’ OR ‘ insulin independent diabetes’ OR ‘ insulin independent diabetes mellitus’ OR ‘ ketosis resistant diabetes mellitus’ OR ‘ maturity onset diabetes’ OR ‘ maturity onset diabetes mellitus’ OR ‘ maturity onset diabetes of the young’ OR ‘ niddm’ OR ‘ non insulin dependent diabetes’ OR ‘ noninsulin dependent diabetes’ OR ‘ noninsulin dependent diabetes mellitus’ OR ‘ type 2 diabetes’ OR ‘ type 2 diabetes mellitus’ OR ‘ type II diabetes’ |
| 10 | 'insulin resistance'/exp OR 'insuline resistance' OR 'glucose intolerance'/exp OR 'glucose intolerant' OR 'insulin resistant' OR 'insuline resistant' OR 'glucose blood level'/exp OR ‘blood glucose’ OR ‘ blood serum glucose’ OR ‘ blood sugar’ OR ‘ glucose, blood’ OR ‘ glucose, plasma’ OR ‘ glycemia’ OR ‘ plasma glucose’ OR ‘ serum glucose’ OR ‘ serum sugar’ OR 'hyperglycemia'/exp OR ‘glucose blood level, elevated’ OR ‘ glycemia, hyper’ OR ‘ hyperglucemia’ OR ‘ hyperglycaemia’ OR ‘ hyperglycemic syndrome’ OR 'insulin'/exp OR ‘ insuline’ OR ‘ insulinum’ OR hyperglycem*.ti,ab OR hyperinsulin*:ti,ab |
| 11 | 'growth, development and aging'/de OR 'growth'/de OR 'body size'/de OR 'body height'/de OR 'adolescent development'/exp OR 'child development'/exp |
| 12 | 'child'/exp OR 'child' OR 'children'/exp OR 'children' OR 'adolescent'/exp OR adolescent OR kid OR kids OR 'young patient' OR boy* OR girl* OR 'young age' OR pediatr* OR paediatr* OR 'child health'/exp OR 'child health' OR child* |
| 13 | #1 AND (#3 OR #4 OR #7 OR #8 OR #9 OR #10 OR #11) AND #12 |
| 15 | 'organisms'/exp NOT 'human'/exp |
| 16 | #13 NOT #15 |

**Cochrane Central Register of Controlled Trials**

**ID Search**

#1 MeSH descriptor: [Fatty Acids] this term only

#2 MeSH descriptor: [Myristic Acids] explode all trees

#3 MeSH descriptor: [Palmitic Acids] explode all trees

#4 MeSH descriptor: [Stearic Acids] explode all trees

#5 MeSH descriptor: [Lauric Acids] explode all trees

#6 MeSH descriptor: [Dietary Fats] this term only

#7 MeSH descriptor: [Diet, Fat-Restricted] explode all trees

#8 saturated* or "myristic" or myristate* or "palmitic" or palmitate* or "stearic" or stearate* or "lauric" or laurate* or "long chain fatty acids" or "very-long chain fatty acids" or "medium chain fatty acids" or dairy fat* or animal fat* or "coconut oil" or "palm oil" or "palm kernel oil" or "peanut oil" or animal fat* or dietary fat* or "reduced fat" or "reducing fat" or "low fat" or "modified fat" or "modifying fat" or "lower fat" or "lowered fat" or "fat intake"

#9 #1 or #2 or #3 or #4 or #5 or #6 or #7 or #8

#10 MeSH descriptor: [Cholesterol] explode all trees

#11 MeSH descriptor: [Lipoproteins] explode all trees

#12 MeSH descriptor: [Hypercholesterolemia] explode all trees

#13 (blood lipid* or "cholesterol" or lipoprotein* or "LDL" or "HDL" or "LDL/HDL" or "total/HDL" or "apolipoprotein(a)" or "apo(a)")

#14 #10 or #11 or #12 or #13

#15 MeSH descriptor: [Triglycerides] explode all trees

#16 MeSH descriptor: [Hypertriglyceridemia] explode all trees

#17 *triglycer* or triacylgly* or "TG/HDL" or hypertriglycer*

#18 #15 or #16 or #17

#19 MeSH descriptor: [Body Weight] explode all trees

#20 MeSH descriptor: [Body Mass Index] explode all trees

#21 MeSH descriptor: [Waist Circumference] explode all trees

#22 MeSH descriptor: [Obesity] explode all trees

#23 MeSH descriptor: [Skinfold Thickness] explode all trees

#24 MeSH descriptor: [Waist-Hip Ratio] explode all trees

#25 MeSH descriptor: [Body Fat Distribution] explode all trees

#26 MeSH descriptor: [Abdominal Fat] explode all trees

#27 "body mass" or "weight" or "weights" or "BMI" or "overweight" or "obesity" or "obese" or "waist circumference" or "waist circumferences" or "waist-hip" or "waist-to-hip" or "body fat" or "body fats" or adipos* or "percentage body fat" or "metabolic syndrome"

#28 #19 or #20 or #21 or #22 or #23 or #24 or #25 or #26 or #27

#29 MeSH descriptor: [Blood Pressure] explode all trees

#30 MeSH descriptor: [Hypertension] explode all trees

#31 blood pressure* or "hypertension" or "systolic" or "diastolic"

#32 #29 or #30 or #31

#33 MeSH descriptor: [Diabetes Mellitus, Type 2] explode all trees

#34 "diabetes" or "MODY" or "NIDDM"

#35 #33 or #34

#36 MeSH descriptor: [Insulin Resistance] explode all trees

#37 MeSH descriptor: [Glucose Intolerance] explode all trees

#38 MeSH descriptor: [Blood Glucose] explode all trees

#39 MeSH descriptor: [Hyperglycemia] explode all trees

#40 "insulin" or "glucose" or hyperglycem* or hyperinsulin* or insulin sensitiv*

#41 #36 or #37 or #38 or #39 or #40

#42 MeSH descriptor: [Growth and Development] this term only

#43 MeSH descriptor: [Growth] this term only

#44 MeSH descriptor: [Body Size] this term only

#45 MeSH descriptor: [Body Height] explode all trees

#46 MeSH descriptor: [Child Development] explode all trees

#47 MeSH descriptor: [Adolescent Development] explode all trees

#48 growth or development

#49 #42 or #43 or #44 or #45 or #46 or #47 or #48

#50 MeSH descriptor: [Child] explode all trees

#51 MeSH descriptor: [Adolescent] explode all trees

#52 MeSH descriptor: [Young Adult] explode all trees

#53 "child" or "children" or "adolescent" or "adolescents" or "adolescence" or "teen" or "teens" or "teenager" or "teenagers" or "youth" or "youths" or "childhood" or "young adult" or "young adults"

#54 #50 or #51 or #52 or #53

#55 MeSH descriptor: [Humans] explode all trees

#56 MeSH descriptor: [Animals] explode all trees

#57 (((#9 and #14) or (#9 and #18) or (#9 and #28) or (#9 and #32) or (#9 and #35) or (#9 and #41) or (#9 and #49)) and #54) not (#56 not #55)

**LILACS**

**Search 1:** (saturated fat) AND (cholesterol OR triglycerides OR LDL OR HDL OR blood lipids) AND (children OR adolescents OR young adults)

**Search 2:** (saturated fat) AND (blood pressure) AND (children OR adolescents OR young adults)

**Search 3:** (saturated fat) AND (diabetes OR insulin resistance) AND (children OR adolescents OR young adults)

**Search 4:** (saturated fat) AND (overweight OR obesity OR weight gain) AND (children OR adolescents OR young adults)

**Search 5:** (saturated fat) AND (growth OR development) AND (children OR adolescents OR young adults)

**WHO International Clinical Trials Registry Platform (ICTRP)**

saturated fat AND cholesterol OR saturated fat AND blood lipids OR saturated fat AND triglycerides OR saturated fat AND blood pressure OR saturated fat AND diabetes OR saturated fat AND insulin resistance OR saturated fat AND weight gain OR saturated fat AND overweight OR saturated fat AND obesity OR saturated fat AND growth OR saturated fat AND development

**Search terms used in the literature review for trans-fatty acids**

**PubMed**

#1 (“Trans Fatty Acids” [MeSH] OR trans fat* [tiab] OR trans-18* [tiab] OR trans-16* [tiab] OR "Linoleic acids, conjugated" [MeSH] OR "conjugated linoleic" [tiab] OR “CLA” [tiab] OR “vaccenic acid” [tiab] OR “vaccenyl” [tiab] OR “vaccenate” [tiab] OR “partially hydrogenated” [tiab] OR “partial hydrogenation” [tiab] OR “Margarine” [MeSH] OR “margarine” [tiab] OR processed food* [tiab] OR animal fat* [tiab] OR ruminant* [tiab] OR rumenic* [tiab])

#2 ("cholesterol" [MeSH] OR "lipoproteins" [MeSH] OR blood lipid* [tiab] OR “cholesterol" [tiab] OR "Hypercholesterolemia"[Mesh] OR lipoprotein* [tiab] OR “LDL” [tiab] OR “HDL” [tiab] OR “LDL/HDL” [tiab] OR “total/HDL” [tiab] OR “apolipoprotein(a)” [tiab] OR “apo(a)” [tiab])

#3 ("triglycerides" [MeSH] OR "Hypertriglyceridemia"[Mesh] OR triglycer* [tiab] OR triacylgly* [tiab] OR “TG/HDL” [tiab] OR hypertriglycer* [tiab])

#4 (("body weight" [MeSH] NOT (“Birth weight” [MeSH] OR “Fetal weight” [MeSH] OR “Thinness” [MeSH])) OR "body mass index" [MeSH] OR "waist circumference" [MeSH] OR "obesity" [MeSH] OR "waist-hip ratio" [MeSH] OR "body fat distribution" [MeSH] OR “Skinfold thickness” [MeSH] OR "Abdominal Fat/drug effects"[Mesh] OR "Abdominal Fat/growth and development" [Mesh] OR “body mass” [tiab] OR "weight” [tiab] OR "weights” [tiab] OR "BMI" [tiab] OR "overweight" OR "obesity" [tiab] OR “obese” OR "waist circumference" [tiab] OR "waist circumferences" OR "waist-hip" [tiab] OR "waist-to-hip" [tiab] OR "body fat" [tiab] OR "body fats" [tiab] OR adipos* [tiab] OR “percentage body fat” [tiab] OR “metabolic syndrome”)

#5 ("blood pressure" [MeSH] OR "hypertension" [MeSH] OR blood pressure* [tiab] OR "hypertension" [tiab] OR "systolic" [tiab] OR "diastolic" [tiab])

#6 ("diabetes mellitus, type 2" [MeSH] OR “diabetes” [tiab] OR "MODY" [tiab] OR "NIDDM" [tiab])

#7 ("insulin resistance" [MeSH] OR "glucose Intolerance" [MeSH] OR "blood glucose" [MeSH] OR "hyperglycemia" [MeSH] OR "insulin" [tiab] OR "glucose" [tiab] OR hyperglycem* [tiab] OR hyperinsulin* [tiab] OR insulin sensitiv* [tiab])

#8 ("Growth and Development" [Mesh:NoExp] OR "Growth" [Mesh:NoExp] OR "Body Size" [Mesh NoExp] OR “Body height” [MeSH] OR "Adolescent Development" [Mesh] OR "Child Development" [Mesh] OR "growth" [tiab] OR "development" [tiab])

#9 ("child"[MeSH] OR "adolescent"[MeSH] OR "young adult"[MeSH] OR "child"[tiab] OR "children"[tiab] OR "adolescent"[tiab] OR "adolescents"[tiab] OR "adolescence"[tiab] OR "teen"[tiab] OR "teens"[tiab] OR "teenager"[tiab] OR "teenagers"[tiab] OR "youth"[tiab] OR "youths"[tiab] OR "childhood"[tiab] OR "young adult"[tiab] OR "young adults"[tiab])

**((#1 AND #2) OR (#1 AND #3) OR (#1 AND #4) OR (#1 AND #5) OR (#1 AND #6) OR (#1 AND #7) OR (#1 AND #8) AND #9) NOT ("animals"[MeSH] NOT "humans"[MeSH])**

**EMBASE**

| **#** | **Searches** |
| --- | --- |
| 1 | 'trans fatty acid'/exp OR 'conjugated linoleic acid'/exp OR 'margarine'/exp OR ‘trans fat’:ti,ab OR ‘trans fats’:ti,ab OR ‘trans 18’:ti,ab OR ‘trans 16’:ti,ab OR ‘conjugated linoleic’:ti,ab OR ‘CLA’:ti,ab OR ‘vaccenic acid’:ab,ti OR ‘vaccenyl’:ti,ab OR ‘vaccenate’:ti,ab OR ‘partially hydrogenated’:ti,ab OR ‘partial hydrogenation’:ti,ab OR ‘margarine’:ti,ab OR ‘processed food’:ti,ab OR ‘processed foods’:ti,ab OR ruminant*:ti,ab OR rumenic*:ti,ab |
| 3 | 'cholesterol'/exp OR ‘ lipoproteins’ OR ‘blood lipids’ OR ‘blood lipid’ OR ‘cholesterol’ OR ‘apolipoprotein’ OR ‘cholesteremia’ OR ‘ cholesterinemia’ OR ‘ cholesterolemia’ OR ‘hypercholesteremia’ OR ‘hypercholesterinaemia’ OR ‘hypercholesterinemia’ OR ‘hypercholesterolaemia’ OR ‘beta lipoprotein’ OR ‘ LDL’ OR ‘HDL’ OR ‘apo a’ OR ‘lipoprotein’ |
| 4 | 'triacylglycerol'/exp OR 'triacylglycerol' OR ‘ triacyl glyceride’ OR ‘ triglyceride’ OR ‘ triglycerides’ OR ‘ tryglyceride’ OR ‘hypertriglyceridaemia’ OR triglycer*:ti,ab OR triacylgly*:ti,ab OR ‘TG/HDL’ OR hypertriglycer*:ti,ab |
| 5 | 'body mass'/exp OR ‘BMI (body mass index)’ OR ‘body ban mass’ OR ‘body mass index’ OR ‘Quetelet index’ OR 'waist circumference'/exp OR ‘adipose tissue hyperplasia’ OR ‘adipositas’ OR ‘ diposity’ OR ‘alimentary obesity’ OR ‘ body weight, excess’ OR ‘fat overload syndrome’ OR ‘nutritional obesity’ OR ‘obesitas’ OR ‘overweight’ OR 'obesity'/exp OR ‘hip to waist ratio’ OR ‘hip waist ratio’ OR ‘waist-hip ratio’ OR ‘waist to hip ratio’ OR 'waist hip ratio'/exp OR ‘adipose tissue distribution’ OR ‘fat tissue distribution’ OR ‘fatty tissue distribution’ OR ‘subcutaneous fat distribution’ OR ‘visceral fat distribution’ OR 'body fat distribution'/exp OR ‘skin fold measurement’ OR ‘skin fold thickness’ OR ‘skin thickness’ OR ‘skinfold measurement’ OR 'skinfold thickness'/exp OR (('abdominal fat'/exp OR ‘abdominal adipose tissue’) AND 'drug effect'/exp) OR ‘body mass’:ti,ab OR ‘weight’:ti,ab OR ‘weights’:ti,ab OR ‘BMI’:ti,ab OR ‘overweight’ OR ‘obesity’:ti,ab OR “obese” OR ‘waist circumference’:ti,ab OR ‘waist circumferences’:ti,ab OR ‘waist-hip’:ti,ab OR ‘waist-to-hip’:ti,ab OR ‘body fat’:ti,ab OR ‘body fats’:ti,ab OR adipos*:ti,ab OR ‘metabolic syndrome’:ti,ab |
| 6 | 'body weight'/exp NOT ( 'birth weight'/exp OR 'fetus weight'/exp) |
| 7 | #5 OR #6 |
| 8 | 'hypertension'/exp OR 'blood pressure'/exp OR 'blood pressure monitoring'/exp OR ‘hypertensive disease’ OR ‘hypertensive effect’ OR ‘hypertensive response’ OR ‘systolic’ OR ‘diastolic’ |
| 9 | 'non insulin dependent diabetes mellitus'/exp OR MODY OR ‘adult onset diabetes’ OR ‘ adult onset diabetes mellitus’ OR ‘ diabetes mellitus type 2’ OR ‘ diabetes mellitus type ii’ OR ‘ diabetes mellitus, non-insulin-dependent’ OR ‘ diabetes mellitus, type 2’ OR ‘ diabetes mellitus, type II’ OR ‘ diabetes mellitus, maturity onset’ OR ‘ diabetes mellitus, non insulin dependent’ OR ‘ diabetes type 2’ OR ‘ diabetes type II’ OR ‘ diabetes, adult onset’ OR ‘ dm 2’ OR ‘ insulin independent diabetes’ OR ‘ insulin independent diabetes mellitus’ OR ‘ ketosis resistant diabetes mellitus’ OR ‘ maturity onset diabetes’ OR ‘ maturity onset diabetes mellitus’ OR ‘ maturity onset diabetes of the young’ OR ‘ niddm’ OR ‘ non insulin dependent diabetes’ OR ‘ noninsulin dependent diabetes’ OR ‘ noninsulin dependent diabetes mellitus’ OR ‘ type 2 diabetes’ OR ‘ type 2 diabetes mellitus’ OR ‘ type II diabetes’ |
| 10 | 'insulin resistance'/exp OR 'insuline resistance' OR 'glucose intolerance'/exp OR 'glucose intolerant' OR 'insulin resistant' OR 'insuline resistant' OR 'glucose blood level'/exp OR ‘blood glucose’ OR ‘ blood serum glucose’ OR ‘ blood sugar’ OR ‘ glucose, blood’ OR ‘ glucose, plasma’ OR ‘ glycemia’ OR ‘ plasma glucose’ OR ‘ serum glucose’ OR ‘ serum sugar’ OR 'hyperglycemia'/exp OR ‘glucose blood level, elevated’ OR ‘ glycemia, hyper’ OR ‘ hyperglucemia’ OR ‘ hyperglycaemia’ OR ‘ hyperglycemic syndrome’ OR 'insulin'/exp OR ‘ insuline’ OR ‘ insulinum’ OR hyperglycem*.ti,ab OR hyperinsulin*:ti,ab |
| 11 | 'growth, development and aging'/de OR 'growth'/de OR 'body size'/de OR 'body height'/de OR 'adolescent development'/exp OR 'child development'/exp |
| 12 | 'child'/exp OR 'child' OR 'children'/exp OR 'children' OR 'adolescent'/exp OR adolescent OR kid OR kids OR 'young patient' OR boy* OR girl* OR 'young age' OR pediatr* OR paediatr* OR 'child health'/exp OR 'child health' OR child* |
| 13 | #1 AND (#3 OR #4 OR #7 OR #8 OR #9 OR #10 OR #11) AND #12 |
| 15 | 'organisms'/exp NOT 'human'/exp |
| 16 | #13 NOT #15 |

**Cochrane Central Register of Controlled Trials**

**ID Search**

#1 MeSH descriptor: [Butter] explode all trees

#2 MeSH descriptor: [Trans Fatty Acids] explode all trees

#3 MeSH descriptor: [Linoleic Acids, Conjugated] explode all trees

#4 MeSH descriptor: [Margarine] explode all trees

#5 trans fat* or trans-18* or trans-16* or "conjugated linoleic" or "CLA" or "vaccenic acid" or "vaccenyl" or "vaccenate" or "partially hydrogenated" or "partial hydrogenation" or "margarine" or processed food* or "butter" or ruminant* or rumenic*

#6 #1 or #2 or #3 or #4 or #5

#7 MeSH descriptor: [Cholesterol] explode all trees

#8 MeSH descriptor: [Lipoproteins] explode all trees

#9 MeSH descriptor: [Hypercholesterolemia] explode all trees

#10 (blood lipid* or "cholesterol" or lipoprotein* or "LDL" or "HDL" or "LDL/HDL" or "total/HDL" or "apolipoprotein(a)" or "apo(a)")

#11 #7 or #8 or #9 or #10

#12 MeSH descriptor: [Triglycerides] explode all trees

#13 MeSH descriptor: [Hypertriglyceridemia] explode all trees

#14 *triglycer* or triacylgly* or "TG/HDL" or hypertriglycer*

#15 #12 or #13 or #14

#16 MeSH descriptor: [Body Weight] explode all trees

#17 MeSH descriptor: [Body Mass Index] explode all trees

#18 MeSH descriptor: [Waist Circumference] explode all trees

#19 MeSH descriptor: [Obesity] explode all trees

#20 MeSH descriptor: [Skinfold Thickness] explode all trees

#21 MeSH descriptor: [Waist-Hip Ratio] explode all trees

#22 MeSH descriptor: [Body Fat Distribution] explode all trees

#23 MeSH descriptor: [Abdominal Fat] explode all trees

#24 "body mass" or "weight" or "weights" or "BMI" or "overweight" or "obesity" or "obese" or "waist circumference" or "waist circumferences" or "waist-hip" or "waist-to-hip" or "body fat" or "body fats" or adipos* or "percentage body fat" or "metabolic syndrome"

#25 #16 or #17 or #18 or #19 or #20 or #21 or #22 or #23 or #24

#26 MeSH descriptor: [Blood Pressure] explode all trees

#27 MeSH descriptor: [Hypertension] explode all trees

#28 blood pressure* or "hypertension" or "systolic" or "diastolic"

#29 #26 or #27 or #28

#30 MeSH descriptor: [Diabetes Mellitus, Type 2] explode all trees

#31 "diabetes" or "MODY" or "NIDDM"

#32 #30 or #31

#33 MeSH descriptor: [Insulin Resistance] explode all trees

#34 MeSH descriptor: [Glucose Intolerance] explode all trees

#35 MeSH descriptor: [Blood Glucose] explode all trees

#36 MeSH descriptor: [Hyperglycemia] explode all trees

#37 "insulin" or "glucose" or hyperglycem* or hyperinsulin* or insulin sensitiv*

#38 #33 or #34 or #35 or #36 or #37

#39 MeSH descriptor: [Growth and Development] this term only

#40 MeSH descriptor: [Growth] this term only

#41 MeSH descriptor: [Body Size] this term only

#42 MeSH descriptor: [Body Height] explode all trees

#43 MeSH descriptor: [Child Development] explode all trees

#44 MeSH descriptor: [Adolescent Development] explode all trees

#45 growth or development

#46 #39 or #40 or #41 or #42 or #43 or #44 or #45

#47 MeSH descriptor: [Child] explode all trees

#48 MeSH descriptor: [Adolescent] explode all trees

#49 MeSH descriptor: [Young Adult] explode all trees

#50 "child" or "children" or "adolescent" or "adolescents" or "adolescence" or "teen" or "teens" or "teenager" or "teenagers" or "youth" or "youths" or "childhood" or "young adult" or "young adults"

#51 #47 or #48 or #49 or #50

#52 MeSH descriptor: [Humans] explode all trees

#53 MeSH descriptor: [Animals] explode all trees

#54 (((#6 and #11) or (#6 and #15) or (#6 and #25) or (#6 and #29) or (#6 and #32) or (#6 and #38) or (#6 and #46)) and #51) not (#53 not #52)

**LILACS**

**Search 1:** (trans fat) AND (cholesterol OR triglycerides OR LDL OR HDL OR blood lipids) AND (children OR adolescents OR young adults)

**Search 2:** (trans fat) AND (blood pressure) AND (children OR adolescents OR young adults)

**Search 3:** (trans fat) AND (diabetes OR insulin resistance) AND (children OR adolescents OR young adults)

**Search 3:** (trans fat) AND (overweight OR obesity OR weight gain) AND (children OR adolescents OR young adults)

**Search 4:** (trans fat) AND (growth OR development) AND (children OR adolescents OR young adults)

**WHO clinical trial registry**

trans fat AND cholesterol OR trans fat AND blood lipids OR trans fat AND triglycerides OR trans fat AND blood pressure OR trans fat AND diabetes OR trans fat AND insulin resistance OR trans fat AND weight gain OR trans fat AND overweight OR trans fat AND obesity OR trans fat AND growth OR trans fat AND development
