# Supplementary material for: Health effects of saturated and trans-fatty acid intake in children and adolescents: Systematic review and meta-analysis
Source: PLoS One. 2017 Nov 17;12(11):e0186672. doi: 10.1371/journal.pone.0186672 (PMC5693282; doi:10.1371/journal.pone.0186672)
Supplement: S4 Table — (DOCX) [file pone.0186672.s004.docx]

**S Table 4**. Notes on multi-timepoint studies

*STRIP*

Various outcomes were reported for the STRIP study over nearly 20 years of follow-up. The effects of the intervention on total cholesterol were reported for each age from 2 to 19 years (starting at 13 months), with the apparent exception of ages 6 and 8. We examined the temporal changes in total cholesterol across each age reported (**Supplemental Figure 8**). The findings were relatively consistent with a mean effect of -0.15 mmol/l ([95%CI: -0.19 to -0.12]; p < 0.001). Standardized mean differences in height (0.03 cm [95%CI: -0.02 to 0.08]; p = 0.21) and body weight (0.03 kg [95%CI: -0.05 to 0.11]; p = 0.48) were also determined though neither was statistically significant (data not shown).

*DISC*

The initial DISC protocol was designed for three years (with outcomes assessed at baseline, year 1 and year 3) however at the end of the study, participants were invited to participate in an extension of the study until 18 years of age. The frequency and nature of the offered intervention changed during the extension period and intervention attendance decreased. Given the uncertainty surrounding the study extension, we only included data only from the final assessment (year 3) of the original three year study.

*Children’s Health Project*

The Children’s Health Project was a one year study that assessed outcomes at baseline, 3months, 6 months and 1 year. Data were reported separately for the 3 month assessment and 6 month and 1 year follow up. LDL cholesterol data from the later follow-ups was reported in graphical form and without a measure of variance thus we used the LDL cholesterol data from the original publication reporting on outcomes at 3 months. Height and weight data at 12 months were reported in sufficient detail to include in the meta-analysis. Total and saturated fat intake data were only published in detail in data the original publication reporting on outcomes at 3 months however graphical information from the publication of outcomes at 12 months showed that significant reductions in total fat intake in the intervention groups compared with the at-risk controls were maintained at 12 months.

*Hendrie 2011*

The study authors assessed outcomes at baseline and at 12 and 24 weeks after initiating intervention. All data was reported in a single publication. We included data from the 24 week assessment.
