# Supplementary material for: Health effects of saturated and trans-fatty acid intake in children and adolescents: Systematic review and meta-analysis
Source: PLoS One. 2017 Nov 17;12(11):e0186672. doi: 10.1371/journal.pone.0186672 (PMC5693282; doi:10.1371/journal.pone.0186672)
Supplement: S5 Table — (DOCX) [file pone.0186672.s005.docx]

**S Table 5**. Characteristics of cohort studies

| **Study ID** | **Citation** | **Number of participants** | **Age (months)** | **Setting** | **Follow-up (months)** | **Exposure assessment method** | **Outcomes measured** | **Correlation with SFA** | **Adjustment for confounders** | **Notes** |
| --- | --- | --- | --- | --- | --- | --- | --- | --- | --- | --- |
| Cowin 2001 | 48 | 389  (55% male) | 18 | UK | 13 | 3-day dietary records | TC, HDL-C, triglycerides, height, weight | TC: *r* = 0.211 (p=0.002, males only);  LDL-C: *r* = 0.174 (p=0.042, males only)  No associations with other variables | Total energy, starch, sugar, non-starch polysaccharides, protein | Results only significant in males. Non-fasted blood samples used so LDL-C results must be viewed with caution. Relatively long period between exposure outcome (cholesterol) assessment. |
| Magarey 2001 | 47 | 143 (45% male at 2 years) – 218 (52% male at 15 years) | 24 | Australia | 13 years | Multiple 3 day weighed food record | Height, weight, BMI, triceps, and subscapular skinfold SD scores | Subscapular SD score: β = 0.011 (p 0.04) | Total energy intake, BMI SD score from previous age, Sex, maternal and paternal BMI | Generalized estimating equations |
| Nicklas 1987 | 46 | 50  (50% male) | 6 | US | 6.5 years | 24-hr dietary recall | Total cholesterol, LDL-C, HDL-C, height, weight, subscapular skinfold | No significant correlations. Significant difference in TC and LDL-C between lower tertile (n=17) and upper tertile (n=14) at 4 years | Not specified | Very small sample size |

SFA, saturated fatty acids; TC, total cholesterol; HDL-C, high-density lipoprotein cholesterol; LDL-C, low-density lipoprotein cholesterol; BMI, body mass index; SD, standard deviati
