# Supplementary material for: Health effects of saturated and trans-fatty acid intake in children and adolescents: Systematic review and meta-analysis
Source: PLoS One. 2017 Nov 17;12(11):e0186672. doi: 10.1371/journal.pone.0186672 (PMC5693282; doi:10.1371/journal.pone.0186672)
Supplement: S6 Table — (DOCX) [file pone.0186672.s006.docx]

**S Table 6.**

**GRADE evidence profile 1**

**Author(s):** Jason Montez and Lisa Te Morenga
**Question:** What is the effect of a reduction in saturated fatty acid intake in children?

**Setting:** General child population

| **Quality assessment** | | | | | | | | | **No. of participants^1^** | | | **Relative effect (95%CI)** | **Quality** | | **Importance** |
| --- | --- | --- | --- | --- | --- | --- | --- | --- | --- | --- | --- | --- | --- | --- | --- |
| **No. of studies** | **Design** | | **Risk of bias** | | **Inconsistency** | **Indirectness** | **Imprecision** | **Other considerations** | **Reduced saturated fat intake** | | **Usual saturated fat intake** |  |  |  |  |
| **Total cholesterol (follow-up 5 weeks – 19 years; units mmol/L; better indicated by lower values)** | | | | | | | | | | | | | | | |
| 7 | RCTs | | no serious  risk of bias^2^ | | no serious inconsistency^3^ | no serious indirectness^4^ | no serious imprecision^5^ | none^6^ | 1265 | | 1107 | **MD 0.16 lower**  (0.25 to 0.07 lower) |  HIGH | | IMPORTANT |
| **LDL cholesterol (follow-up 5 weeks – 19 years; units mmol/L; better indicated by lower values)** | | | | | | | | | | | | | | | |
| 7 | RCTs | | no serious  risk of bias^7^ | | no serious inconsistency^8^ | no serious indirectness^4^ | no serious imprecision^5^ | none^6^ | 1098 | | 950 | **MD 0.13 lower**  (0.22 to 0.03 lower) |  HIGH | | CRITICAL |
| **HDL cholesterol (follow-up 5 weeks – 19 years; units mmol/L; better indicated by higher values)** | | | | | | | | | | | | | | | |
| 6 | RCTs | | no serious  risk of bias^9^ | | no serious inconsistency^10^ | no serious indirectness^4^ | no serious imprecision^11^ | none^6^ | 924 | 863 | | **MD 0.00 lower**  (0.02 lower to 0.02 higher) |  HIGH | | IMPORTANT |
| **Serum triglyceride (follow-up 5 weeks – 19 years; units mmol/L; better indicated by lower values)** | | | | | | | | | | | | | | | |
| 6 | RCTs | | no serious  risk of bias^9^ | | no serious inconsistency^12^ | no serious indirectness^4^ | serious imprecision^13^ | none^6^ | 924 | 863 | | **MD 0.02 lower**  (0.06 lower to 0.01 higher) |  MODERATE | | IMPORTANT |
| **Apolipoprotein A1 (follow-up 28 weeks – 19 years; units mg/dL; better indicated by higher values)** | | | | | | | | | | | | | | | |
| 3 | RCTs | | no serious  risk of bias^14^ | | no serious inconsistency^15^ | no serious indirectness^4^ | serious imprecision^9^ | none^6^ | 409 | 369 | | **MD 1.03 lower**  (3.95 lower to 1.90 higher) |  MODERATE | | IMPORTANT |
| **Apolipoprotein B (follow-up 28 weeks – 19 years; units mg/dL; better indicated by lower values)** | | | | | | | | | | | | | | | |
| 3 | | RCTs | | no serious risk of bias^14^ | serious inconsistency^16^ | no serious indirectness^4^ | serious imprecision^9^ | none^6^ | 409 | 369 | | **MD 1.25 lower**  (6.26 lower to 3.76 higher) |  LOW | IMPORTANT | |
| **Systolic blood pressure (follow-up 12 weeks – 14 years; units mmHg; better indicated by lower values)** | | | | | | | | | | | | | | | |
| 2 | | RCTs | | no serious risk of bias^17^ | no serious inconsistency^18^ | no serious indirectness^19^ | serious imprecision^9^ | none^6^ | 549 | 557 | | **MD 0.68 lower**  (1.71 lower to 0.35 higher) |  MODERATE | CRITICAL | |
| **Diastolic blood pressure (follow-up 12 weeks – 14 years; units mmHg; better indicated by lower values)** | | | | | | | | | | | | | | | |
| 2 | | RCTs | | no serious risk of bias^17^ | no serious inconsistency^18^ | no serious indirectness^19^ | no serious imprecision^5^ | none^6^ | 549 | 557 | | **MD 1.45 lower**  (2.34 lower to 0.56 lower) |  HIGH | CRITICAL | |
| **Body weight (follow-up 5 weeks – 14 years; units SD; better indicated by lower values)** | | | | | | | | | | | | | | | |
| 4 | | RCTs | | no serious risk of bias^20^ | no serious inconsistency^18^ | no serious indirectness^21^ | no serious imprecision^12^ | none^6^ | 797 | 756 | | **SMD 0.03 lower**  (0.13 lower to 0.07 higher) |  HIGH | CRITICAL | |
| **Height (follow-up 12 weeks – 14 years; units SD; better indicated by higher values)** | | | | | | | | | | | | | | | |
| 3 | | RCTs | | no serious risk of bias^20^ | no serious inconsistency^22^ | no serious indirectness^21^ | no serious imprecision^12^ | none^6^ | 664 | 623 | | **SMD 0.09 higher**  (0.03 lower to 0.21 higher) |  HIGH | IMPORTANT | |
| **BMI (follow-up 24 weeks – 19 years; units kg/m^2^; better indicated by lower values)** | | | | | | | | | | | | | | | |
| 3 | | RCTs | | no serious risk of bias^17^ | no serious inconsistency^18^ | no serious indirectness^21^ | no serious imprecision^12^ | none^6^ | 590 | 599 | | **MD 0.10 lower**  (0.32 lower to 0.12 higher) |  HIGH | CRITICAL | |
| **Waist circumference (follow-up 24 weeks – 19 years; units cm; better indicated by lower values)** | | | | | | | | | | | | | | | |
| 2 | | RCTs | | no serious risk of bias^23^ | no serious inconsistency^18^ | no serious indirectness^21^ | serious imprecision^13^ | none^6^ | 276 | 300 | | **MD 0.20 lower**  (1.38 lower to 0.98 higher) |  MODERATE | CRITICAL | |
| **Insulin resistance (follow-up 18 years; measured as HOMA-IR^24^, unitless; better indicated by lower values)** | | | | | | | | | | | | | | | |
| 1 | | RCTs | | serious^25^ | no serious inconsistency^26^ | no serious indirectness^27^ | no serious imprecision^28^ | none^6^ | 245 | 275 | | **MD 7.5% lower**  *p* = 0.0051 |  MODERATE | CRITICAL | |
| **Adverse effects** | | | | | | | | | | | | | | | |
| 2 | | Outcomes reported varied across studies that reported adverse events; not suitable for pooling^29^ | | | | | | | | | | | | | |

CI, confidence interval; RCTs, randomized controlled trials; MD, mean difference; SD, standard deviation; SMD, standardized mean difference; LDL, low-density lipoprotein; HDL, high-density lipoprotein; BMI, body mass index; HOMA-IR, homeostasis model of insulin resistance

^1^ Participants in crossover trials are counted in both the reduced and usual saturated fatty acid (SFA) intake groups.

^2^ One study (Healthy Start) was at high risk of bias in terms of randomization (*33,34*): intervention centres were randomised to treatment 1 or 2, but control groups were selected on the basis that these centres were unable to modify the food service as they received all food from school district procurement system. This may reflect differences in centre characteristics that could influence child health outcomes. One study (DISC) was at high risk of bias in terms of systematic difference in care (*24-27*): regularly scheduled dietary counselling provided to intervention group but no equivalent time with study personnel provided to control group. One study (STRIP) was at high risk of bias in terms of systematic difference in care (*37-45*): dietary counselling was provided to intervention group 2-4 times per year, while control group received basic health education sessions 1-2 times per year. One study (Children’s Health Project) did not report total cholesterol, HDL-cholesterol or triglyceride outcomes even though they reported that this was measured (*29-30*) which could lead to reporting bias for these outcomes. Only one study (Zhu 2003) was at high risk of bias in several areas and thus considered to be at high risk of bias overall (*31*), however results of sensitivity analyses in which this study is removed do not differ significantly from original analyses for most outcomes. Low risk of bias overall.

^3^ I^2^ = 64% indicating a significant level of heterogeneity, however, point estimates are similar and virtually all of the heterogeneity can be explained by the two crossover studies (*32,35*) which achieved greater contrast in SFA intake between control and intervention children than the other studies.

^4^ All studies were conducted in the population of interest, all comparisons were made directly to an appropriate control group and all outcomes are priority outcomes that were decided upon prior to initiating the review. Blood lipids are indirect markers of CVD risk, however, cardiovascular events are rare in children and adolescents. LDL cholesterol is a well-accepted marker of CVD risk in adults: a large meta-analysis of statin therapy RCTs conducted in adults demonstrated a more than 20% reduction in risk of cardiovascular events (coronary death, non-fatal myocardial infarction, coronary revascularization and ischaemic stroke) per 1.0mmol/L reduction in LDL cholesterol (CTT collaboration 2010). Risk of CVD was increased by 6% in men and 12% in women for each 0.2 mmol/l increase in triglyceride concentrations in a meta-analysis of prospective population studies (Hokanson and Austin 1996).

^5^ The 95%CI does not cross threshold of important benefit or harm.

^6^ Too few studies to reliably assess publication bias (<10 studies).

^7^ One study (DISC) was at high risk of bias in terms of systematic difference in care (*24-27*): regularly scheduled dietary counselling provided to intervention group but no equivalent time with study personnel provided to control group. One study (STRIP) was at high risk of bias in terms of systematic difference in care (*37-45*): dietary counselling was provided to intervention group 2-4 times per year, while control group received basic health education sessions 1-2 times per year. One study (Children’s Health Project) was at high risk of bias in terms of systematic difference in care (*28-30*): dietary counselling provided to intervention group but no equivalent time with study personnel provided to control group. Only one study (Zhu 2003) was at high risk of bias in several areas and thus considered to be at high risk of bias overall (*31*), however results of sensitivity analyses in which this study is removed do not differ significantly from original analyses for most outcomes. Low risk of bias overall.

^8^ I^2^ = 77% indicating a high level of heterogeneity, however, most of the heterogeneity can be explained by the two crossover studies (*32,35*) which achieved greater contrast in SFA intake between control and intervention children than the other studies.

^9^ One study (DISC) was at high risk of bias in terms of systematic difference in care (*24-37*): regularly scheduled dietary counselling provided to intervention group but no equivalent time with study personnel provided to control group. One study (STRIP) was at high risk of bias in terms of systematic difference in care (*37-45*): dietary counselling was provided to intervention group 2-4 times per year, while control group received basic health education sessions 1-2 times per year. One study (Children’s Health Project) did not report total cholesterol, HDL-cholesterol or triglyceride outcomes even though they reported that this was measured (*28-30*) which could lead to reporting bias for these outcomes. Only one study (Zhu 2003) was at high risk of bias in several areas and thus considered to be at high risk of bias overall (*31*), however results of sensitivity analyses in which this study is removed do not differ significantly from original analyses for most outcomes. Low risk of bias overall.

^10^ I^2^ = 23%; all 95% CIs overlap.

^11^ The 95%CI crosses zero and does not cross threshold of important benefit or harm and is therefore considered a precise estimate of no effect and not downgraded for serious imprecision.
^12^ I^2^ = 20%; all 95% CIs overlap.

^13^ The 95%CI may cross threshold of important benefit or harm and is therefore downgraded for serious imprecision.

^14^ One study (STRIP) was at high risk of bias in terms of systematic difference in care (*37-45*): dietary counselling was provided to intervention group 2-4 times per year, while control group received basic health education sessions 1-2 times per year. One study (Zhu 2003) was at high risk of bias in several areas and thus considered to be at high risk of bias overall (*31*), however results of sensitivity analyses in which this study is removed do not differ significantly from original analyses for most outcomes. Low risk of bias overall.

^15^ I^2^ = 7%; all 95% CIs overlap.

^16^ I^2^ = 70% indicating a high level of heterogeneity.

^17^ One study (DISC) was at high risk of bias in terms of systematic difference in care (*24-27*): regularly scheduled dietary counselling provided to intervention group but no equivalent time with study personnel provided to control group. One study (STRIP) was also at high risk of bias in terms of systematic difference in care (*37-45*): dietary counselling was provided to intervention group 2-4 times per year, while control group received basic health education sessions 1-2 times per year. Low risk of bias overall.

^18^ I^2^ = 0%; all 95% CIs overlap.

^19^ All studies were conducted in the population of interest, all comparisons were made directly to an appropriate control group and all outcomes are priority outcomes that were decided upon prior to initiating the review. SBP and DBP are indirect markers of CVD risk, however, cardiovascular events are rare in children and adolescents. A meta-analysis (Neal et al. 2000) suggested that modest reductions in blood pressure, (range 3/1 to 6/4 mm Hg SBP/DBP) over the long-term, were associated with reductions in the risk of stroke, coronary heart disease, cardiovascular events and mortality on the order of 20-30%.

^20^ One study (DISC) was at high risk of bias in terms of systematic difference in care (*24-27*): regularly scheduled dietary counselling provided to intervention group but no equivalent time with study personnel provided to control group. One study (STRIP) was at high risk of bias in terms of systematic difference in care (*37-45*): dietary counselling was provided to intervention group 2-4 times per year, while control group received basic health education sessions 1-2 times per year. One study (Children’s Health Project) did not report total cholesterol, HDL-cholesterol or triglyceride outcomes even though they reported that this was measured (*28-30*) which could lead to reporting bias for these outcomes. Low risk of bias overall.

^21^ All studies were conducted in the population of interest, all comparisons were made directly to an appropriate control group and all outcomes are priority outcomes that were decided upon prior to initiating the review. Height and body weight are direct measures of growth. BMI measurements are generally accepted measures of adiposity, though utility in children may be less clear. Waist circumference is an indirect marker of weight gain, overweight and obesity risk. As such, the clinical significance of a reduction or increase in waist circumference in children is unclear.

^22^ I^2^ = 11%; all 95% CIs overlap.

^23^ One study (STRIP) was at high risk of bias in terms of systematic difference in care (*37-45*): dietary counselling was provided to intervention group 2-4 times per year, while control group received basic health education sessions 1-2 times per year. Low risk of bias overall.

^24^ HOMA-IR is a composite measure of insulin resistance incorporating both glucose and insulin as follows: HOMA-IR = fasting glucose(mmol/L) x fasting insulin(mU/L) / 22.5.

^25^ Confounding by dietary fibre intakes which were higher in intervention children than in control children. Dietary fibre was significantly associated with HOMA-IR in girls.

^26^ Only one study included.

^27^ All studies were conducted in the population of interest, all comparisons were made directly to an appropriate control group and all outcomes are priority outcomes that were decided upon prior to initiating the review. Insulin resistance is a strong predictor of type 2 diabetes risk.

^28^ One study included with a small *p* value.

^29^ In addition to no observed effects on growth (i.e. height and weight), there was no evidence of adverse effects of reducing SFA intake in children on micronutrient intakes, cognitive development or sexual maturation in the two studies reporting these outcomes.

**References** (not cited in article)

Cholesterol Treatment Trialists’ (CTT) Collaboration, Baigent C, Blackwell L, Emberson J, Holland LE, Reith C, Bhala N, Peto R, Barnes EH, Keech A, Simes J, Collins R. Efficacy and safety of more intensive lowering of LDL cholesterol: a meta-analysis of data from 170,000 participants in 26 randomised trials.

Lancet. 2010;376(9753):1670-81.

Hokanson JE, Austin MA. Plasma triglyceride level is a risk factor for cardiovascular disease independent of high-density lipoprotein cholesterol level: a meta-analysis of population-based prospective studies. Journal of cardiovascular risk. Apr 1996;3(2):213-9.

Neal B, MacMahon S, Chapman N; Blood Pressure Lowering Treatment Trialists' Collaboration. Effects of ACE inhibitors, calcium antagonists, and other blood-pressure-lowering drugs: results of prospectively designed overviews of randomised trials. Blood Pressure Lowering Treatment Trialists' Collaboration. Lancet. 2000;356(9246):1955-64.

GRADE evidence profile 2

**Author(s):** Jason Montez and Lisa Te Morenga
**Question:** What is the effect of a reduction in saturated fatty acid intake in children with intakes greater than 10% of total energy intake?^1^

**Setting:** General child population

| **Quality assessment** | | | | | | | | | **No. of participants^2^** | | | **Relative effect (95%CI)** | **Quality** | | **Importance** |
| --- | --- | --- | --- | --- | --- | --- | --- | --- | --- | --- | --- | --- | --- | --- | --- |
| **No. of studies** | **Design** | | **Risk of bias** | | **Inconsistency** | **Indirectness** | **Imprecision** | **Other considerations** | **Reduced saturated fat intake** | | **Usual saturated fat intake** |  |  |  |  |
| **Total cholesterol (follow-up 5 weeks – 19 years; units mmol/L; better indicated by lower values)** | | | | | | | | | | | | | | | |
| 6 | RCTs | | no serious  risk of bias^3^ | | no serious inconsistency^4^ | no serious indirectness^5^ | no serious imprecision^6^ | none^7^ | 1145 | | 1067 | **MD 0.18 lower**  (0.28 to 0.09 lower) |  HIGH | | IMPORTANT |
| **LDL cholesterol (follow-up 5 weeks – 19 years; units mmol/L; better indicated by lower values)** | | | | | | | | | | | | | | | |
| 6 | RCTs | | no serious  risk of bias^8^ | | no serious inconsistency^9^ | no serious indirectness^5^ | no serious imprecision^6^ | none^7^ | 939 | | 905 | **MD 0.16 lower**  (0.25 to 0.08 lower) |  HIGH | | CRITICAL |
| **HDL cholesterol (follow-up 5 weeks – 19 years; units mmol/L; better indicated by higher values)** | | | | | | | | | | | | | | | |
| 5 | RCTs | | no serious  risk of bias^10^ | | no serious inconsistency^11^ | no serious indirectness^5^ | no serious imprecision^12^ | none^7^ | 804 | 823 | | **MD 0.00 lower**  (0.01 lower to 0.02 higher) |  HIGH | | IMPORTANT |
| **Serum triglyceride (follow-up 5 weeks – 19 years; units mmol/L; better indicated by lower values)** | | | | | | | | | | | | | | | |
| 5 | RCTs | | no serious  risk of bias^10^ | | no serious inconsistency^13^ | no serious indirectness^5^ | serious imprecision^14^ | none^7^ | 804 | 823 | | **MD 0.03 lower**  (0.07 lower to 0.02 higher) |  MODERATE | | IMPORTANT |
| **Apolipoprotein A1 (follow-up 28 weeks – 19years; units mg/dL; better indicated by higher values)** | | | | | | | | | | | | | | | |
| 2 | RCTs | | no serious  risk of bias^15^ | | no serious inconsistency^11^ | no serious indirectness^5^ | serious imprecision^14^ | none^7^ | 289 | 329 | | **MD 0.35 higher**  (3.30 lower to 4.01 higher) |  MODERATE | | IMPORTANT |
| **Apolipoprotein B (follow-up 28 weeks – 19 years; units mg/dL; better indicated by lower values)** | | | | | | | | | | | | | | | |
| 2 | | RCTs | | no serious risk of bias^15^ | no serious inconsistency^11^ | no serious indirectness^5^ | no serious imprecision^6^ | none^7^ | 289 | 329 | | **MD 4.06 lower**  (7.02 to 1.10 lower) |  HIGH | IMPORTANT | |
| **Systolic blood pressure (follow-up 12 weeks – 14 years; units mmHg; better indicated by lower values)** | | | | | | | | | | | | | | | |
| 2 | | RCTs | | no serious risk of bias^16^ | no serious inconsistency^11^ | no serious indirectness^17^ | serious imprecision^14^ | none^7^ | 549 | 557 | | **MD 0.68 lower**  (1.71 lower to 0.35 higher) |  MODERATE | CRITICAL | |
| **Diastolic blood pressure (follow-up 12 weeks – 14 years; units mmHg; better indicated by lower values)** | | | | | | | | | | | | | | | |
| 2 | | RCTs | | no serious risk of bias^16^ | no serious inconsistency^11^ | no serious indirectness^17^ | no serious imprecision^6^ | none^7^ | 549 | 557 | | **MD 1.45 lower**  (2.34 to 0.56 lower) |  HIGH | CRITICAL | |
| **Body weight (follow-up 5 weeks – 14 years; units SD; better indicated by lower values)** | | | | | | | | | | | | | | | |
| 4 | | RCTs | | no serious risk of bias^18^ | no serious inconsistency^11^ | no serious indirectness^19^ | no serious imprecision^12^ | none^5^ | 797 | 756 | | **SMD 0.03 lower**  (0.13 lower to 0.07 higher) |  HIGH | CRITICAL | |
| **Height (follow-up 12 weeks – 14 years; units SD; better indicated by higher values)** | | | | | | | | | | | | | | | |
| 3 | | RCTs | | no serious risk of bias^18^ | no serious inconsistency^20^ | no serious indirectness^19^ | no serious imprecision^12^ | none | 664 | 623 | | **SMD 0.09 higher**  (0.03 lower to 0.21 higher) |  HIGH | IMPORTANT | |
| **BMI (follow-up 24 weeks – 19 years; units kg/m^2^; better indicated by lower values)** | | | | | | | | | | | | | | | |
| 3 | | RCTs | | no serious risk of bias^16^ | no serious inconsistency^11^ | no serious indirectness^19^ | no serious imprecision^12^ | none | 590 | 599 | | **MD 0.10 lower**  (0.32 lower to 0.12 higher) |  HIGH | CRITICAL | |
| **Waist circumference (follow-up 24 weeks – 19 years; units cm; better indicated by lower values)** | | | | | | | | | | | | | | | |
| 2 | | RCTs | | no serious risk of bias^15^ | no serious inconsistency^11^ | no serious indirectness^19^ | serious imprecision^14^ | none | 276 | 300 | | **MD 0.20 lower**  (1.38 lower to 0.98 higher) |  MODERATE | CRITICAL | |
| **Insulin resistance (follow-up 18 years; measured as HOMA-IR^21^, unitless; better indicated by lower values)** | | | | | | | | | | | | | | | |
| 1 | | RCTs | | serious^22^ | no serious inconsistency^23^ | no serious indirectness^24^ | no serious imprecision^25^ | none | 245 | 275 | | **MD 7.5% lower**  *p* = 0.0051 |  MODERATE | CRITICAL | |
| **Adverse effects** | | | | | | | | | | | | | | | |
| 2 | | Outcomes reported varied across studies that reported adverse events; not suitable for pooling^26^ | | | | | | | | | | | | | |

CI, confidence interval; RCTs, randomized controlled trials; MD, mean difference; SMD, standardized mean difference; LDL, low-density lipoprotein; HDL, high-density lipoprotein; BMI, body mass index; HOMA-IR, homeostasis model of insulin resistance

^1^ Only studies that included a control group consuming greater than 10% of total energy intake as saturated fatty acids (SFA) were included in this analysis.

^2^ Participants in crossover trials are counted in both the reduced and usual SFA intake groups.

^3^ One study (Healthy Start) was at high risk of bias in terms of randomization (*33,34*): intervention centres were randomised to treatment 1 or 2, but control groups were selected on the basis that these centres were unable to modify the food service as they received all food from school district procurement system. This may reflect differences in centre characteristics that could influence child health outcomes. One study (DISC) was at high risk of bias in terms of systematic difference in care (*24-27*): regularly scheduled dietary counselling provided to intervention group but no equivalent time with study personnel provided to control group. One study (STRIP) was at high risk of bias in terms of systematic difference in care (*37-45*): dietary counselling was provided to intervention group 2-4 times per year, while control group received basic health education sessions 1-2 times per year. One study (Children’s Health Project) did not report total cholesterol, HDL-cholesterol or triglyceride outcomes even though they reported that this was measured (*28-30*) which could lead to reporting bias for these outcomes. Low risk of bias overall.

^4^ I^2^ = 64% indicating a significant level of heterogeneity, however, point estimates are similar and virtually all of the heterogeneity can be explained by the two crossover studies (*32,35*) which achieved greater contrast in SFA intake between control and intervention children than the other studies.

^5^ All studies were conducted in the population of interest, all comparisons were made directly to an appropriate control group and all outcomes are priority outcomes that were decided upon prior to initiating the review. Blood lipids are indirect markers of CVD risk, however, cardiovascular events are rare in children and adolescents. LDL cholesterol is a well-accepted marker of CVD risk in adults: a large meta-analysis of statin therapy RCTs conducted in adults demonstrated a more than 20% reduction in risk of cardiovascular events (coronary death, non-fatal myocardial infarction, coronary revascularization and ischaemic stroke) per 1.0mmol/L reduction in LDL cholesterol (CTT collaboration 2010). Risk of CVD was increased by 6% in men and 12% in women for each 0.2 mmol/l increase in triglyceride concentrations in a meta-analysis of prospective population studies (Hokanson and Austin 1996).

^6^ The 95%CI does not cross threshold of important benefit or harm.

^7^ Too few studies to reliably assess publication bias (<10 studies).

^8^ One study (DISC) was at high risk of bias in terms of systematic difference in care (*24-27*): regularly scheduled dietary counselling provided to intervention group but no equivalent time with study personnel provided to control group. One study (STRIP) was at high risk of bias in terms of systematic difference in care (*37-45*): dietary counselling was provided to intervention group 2-4 times per year, while control group received basic health education sessions 1-2 times per year. One study (Children’s Health Project) was at high risk of bias in terms of systematic difference in care (*28-30*): dietary counselling provided to intervention group but no equivalent time with study personnel provided to control group. Low risk of bias overall.

^9^ I^2^ = 67% indicating a significant level of heterogeneity, however, most of the heterogeneity can be explained by the two crossover studies (*32,35*) which achieved greater contrast in SFA intake between control and intervention children than the other studies.

^10^ One study (DISC) was at high risk of bias in terms of systematic difference in care (*24-27*): regularly scheduled dietary counselling provided to intervention group but no equivalent time with study personnel provided to control group. One study (STRIP) was at high risk of bias in terms of systematic difference in care (*37-45*): dietary counselling was provided to intervention group 2-4 times per year, while control group received basic health education sessions 1-2 times per year. One study (Children’s Health Project) did not report total cholesterol, HDL-cholesterol or triglyceride outcomes even though they reported that this was measured (*28-30*) which could lead to reporting bias for these outcomes. Low risk of bias overall.

^11^ I^2^ = 0%; all 95% CIs overlap.

^12^ The 95%CI crosses zero and does not cross threshold of important benefit or harm and is therefore considered a precise estimate of no effect and not downgraded for serious imprecision.
^13^ I^2^ = 36%; all 95% CIs overlap.

^14^ The 95%CI may cross threshold of important benefit or harm and is therefore downgraded for serious imprecision.

^15^ One study (STRIP) was at high risk of bias in terms of systematic difference in care (*37-45*): dietary counselling was provided to intervention group 2-4 times per year, while control group received basic health education sessions 1-2 times per year. Low risk of bias overall.

^16^ One study (DISC) was at high risk of bias in terms of systematic difference in care (*24-27*): regularly scheduled dietary counselling provided to intervention group but no equivalent time with study personnel provided to control group. One study (STRIP) was also at high risk of bias in terms of systematic difference in care (*37-45*): dietary counselling was provided to intervention group 2-4 times per year, while control group received basic health education sessions 1-2 times per year. Low risk of bias overall.

^17^ All studies were conducted in the population of interest, all comparisons were made directly to an appropriate control group and all outcomes are priority outcomes that were decided upon prior to initiating the review. SBP and DBP are indirect markers of CVD risk, however, cardiovascular events are rare in children and adolescents. A meta-analysis (Neal et al. 2000) suggested that modest reductions in blood pressure, (range 3/1 to 6/4 mm Hg SBP/DBP) over the long-term, were associated with reductions in the risk of stroke, coronary heart disease, cardiovascular events and mortality on the order of 20-30%.

^18^ One study (DISC) was at high risk of bias in terms of systematic difference in care (*24-27*): regularly scheduled dietary counselling provided to intervention group but no equivalent time with study personnel provided to control group. One study (STRIP) was at high risk of bias in terms of systematic difference in care (*37-45*): dietary counselling was provided to intervention group 2-4 times per year, while control group received basic health education sessions 1-2 times per year. One study (Children’s Health Project) did not report total cholesterol, HDL-cholesterol or triglyceride outcomes even though they reported that this was measured (*28-30*) which could lead to reporting bias for these outcomes. Low risk of bias overall.

^19^ All studies were conducted in the population of interest, all comparisons were made directly to an appropriate control group and all outcomes are priority outcomes that were decided upon prior to initiating the review. Height and body weight are direct measures of growth. BMI measurements are generally accepted measures of adiposity, though utility in children may be less clear. Waist circumference is an indirect marker of weight gain, overweight and obesity risk. As such, the clinical significance of a reduction or increase in waist circumference in children is unclear.

^20^ I^2^ = 11%; all 95% CIs overlap.

^21^ HOMA-IR is a composite measure of insulin resistance incorporating both glucose and insulin as follows: HOMA-IR = fasting glucose(mmol/L) x fasting insulin(mU/L) / 22.5.

^22^ Confounding by dietary fibre intakes which were higher in intervention children than in control children. Dietary fibre was significantly associated with HOMA-IR in girls.

^23^ Only one study included.

^24^ All studies were conducted in the population of interest, all comparisons were made directly to an appropriate control group and all outcomes are priority outcomes that were decided upon prior to initiating the review. Insulin resistance is a strong predictor of type 2 diabetes risk.

^25^ One study included with a small *p* value.

^26^ In addition to no observed effects on growth (i.e. height and weight), there was no evidence of adverse effects of reducing SFA intake in children on micronutrient intakes, cognitive development or sexual maturation in the two studies reporting these outcomes.

**References** (not cited in article)

Cholesterol Treatment Trialists’ (CTT) Collaboration, Baigent C, Blackwell L, Emberson J, Holland LE, Reith C, Bhala N, Peto R, Barnes EH, Keech A, Simes J, Collins R. Efficacy and safety of more intensive lowering of LDL cholesterol: a meta-analysis of data from 170,000 participants in 26 randomised trials.

Lancet. 2010;376(9753):1670-81.

Hokanson JE, Austin MA. Plasma triglyceride level is a risk factor for cardiovascular disease independent of high-density lipoprotein cholesterol level: a meta-analysis of population-based prospective studies. Journal of cardiovascular risk. Apr 1996;3(2):213-9.

Neal B, MacMahon S, Chapman N; Blood Pressure Lowering Treatment Trialists' Collaboration. Effects of ACE inhibitors, calcium antagonists, and other blood-pressure-lowering drugs: results of prospectively designed overviews of randomised trials. Blood Pressure Lowering Treatment Trialists' Collaboration. Lancet. 2000;356(9246):1955-64.

**GRADE evidence profile 3**

**Author(s):** Jason Montez and Lisa Te Morenga
**Question:** What is the effect of a reduction in saturated fatty acid intake in children to less than 10% of total energy intake?^1^

**Setting:** General child population

| **Quality assessment** | | | | | | | **No. of participants^2^** | | | **Relative effect (95%CI)** | **Quality** | **Importance** |
| --- | --- | --- | --- | --- | --- | --- | --- | --- | --- | --- | --- | --- |
| **No. of studies** | **Design** | **Risk of bias** | **Inconsistency** | **Indirectness** | **Imprecision** | **Other considerations** | **Reduced saturated fat intake** | | **Usual saturated fat intake** |  |  |  |
| **Total cholesterol (follow-up 5 weeks; units mmol/L; better indicated by lower values)** | | | | | | | | | | | | |
| 1 | RCTs | no serious  risk of bias^3^ | no serious inconsistency^4^ | no serious indirectness^5^ | no serious imprecision^6^ | none^7^ | 134 | | 134 | **MD 0.29 lower**  (0.40 to 0.18 lower) |  HIGH | IMPORTANT |
| **LDL cholesterol (follow-up 5 weeks; units mmol/L; better indicated by lower values)** | | | | | | | | | | | | |
| 1 | RCTs | no serious  risk of bias^3^ | no serious inconsistency^4^ | no serious indirectness^5^ | no serious imprecision^6^ | none^7^ | 134 | | 134 | **MD 0.29 lower**  (0.38 to 0.20 lower) |  HIGH | CRITICAL |
| **HDL cholesterol (follow-up 5 weeks; units mmol/L; better indicated by higher values)** | | | | | | | | | | | | |
| 1 | RCTs | no serious  risk of bias^3^ | no serious inconsistency^4^ | no serious indirectness^5^ | no serious imprecision^8^ | none^7^ | 134 | 134 | | **MD 0.00 lower**  (0.03 lower to 0.03 higher) |  HIGH | IMPORTANT |
| **Serum triglyceride (follow-up 5 weeks; units mmol/L; better indicated by lower values)** | | | | | | | | | | | | |
| 1 | RCTs | no serious  risk of bias^3^ | no serious inconsistency^4^ | no serious indirectness^5^ | serious imprecision^9^ | none^7^ | 134 | 134 | | **MD 0.02 lower**  (0.07 lower to 0.03 higher) |  MODERATE | IMPORTANT |
| **Apolipoprotein A1** | | | | | | | | | | | | |
| 0 | No studies identified reporting this outcome | | | | | | | | | | | |
| **Apolipoprotein B** | | | | | | | | | | | | |
| 0 | No studies identified reporting this outcome | | | | | | | | | | | |
| **Systolic blood pressure** | | | | | | | | | | | | |
| 0 | No studies identified reporting this outcome | | | | | | | | | | | |
| **Diastolic blood pressure** | | | | | | | | | | | | |
| 0 | No studies identified reporting this outcome | | | | | | | | | | | |
| **Body weight (follow-up 5weeks; units kg; better indicated by lower values)** | | | | | | | | | | | | |
| 1 | RCTs | no serious  risk of bias^3^ | no serious inconsistency^4^ | no serious indirectness^10^ | serious imprecision^9^ | none^5^ | 134 | 134 | | **MD 0.20 lower**  (0.63 lower to 0.23 higher) |  MODERATE | IMPORTANT |
| **Height** | | | | | | | | | | | | |
| 0 | No studies identified reporting this outcome | | | | | | | | | | | |
| **BMI** | | | | | | | | | | | | |
| 0 | No studies identified reporting this outcome | | | | | | | | | | | |
| **Waist circumference** | | | | | | | | | | | | |
| 0 | No studies identified reporting this outcome | | | | | | | | | | | |
| **Insulin resistance** | | | | | | | | | | | | |
| 0 | No studies identified reporting this outcome | | | | | | | | | | | |
| **Adverse effects** | | | | | | | | | | | | |
| 0 | No studies identified reporting this outcome | | | | | | | | | | | |

CI, confidence interval; RCTs, randomized controlled trials; MD, mean difference; SMD, standardized mean difference; LDL, low-density lipoprotein; HDL, high-density lipoprotein; BMI, body mass index

^1^ Studies included in this analysis were limited to those with an intervention group achieving less than 10% of total energy intake as saturated fatty acids (SFA). One study (Zhu 2003) was excluded as though the intervention group achieved SFA intake of less than 10% of total energy intake, the control group was reported as having a starting intake of well below 10% which was further reduced during the study, raising serious questions about the accurate recording and/or reporting of dietary intake data.

^2^ Participants in this crossover trial are counted in both the reduced and usual SFA intake groups.

^3^ No serious risk of bias in the included study (Denke 2000).

^4^ Only one study included.

^5^ The study was conducted in the population of interest, all comparisons were made directly to an appropriate control group and all outcomes are priority outcomes that were decided upon prior to initiating the review. Blood lipids are indirect markers of CVD risk, however, cardiovascular events are rare in children and adolescents. LDL cholesterol is a well-accepted marker of CVD risk in adults: a large meta-analysis of statin therapy RCTs conducted in adults demonstrated a more than 20% reduction in risk of cardiovascular events (coronary death, non-fatal myocardial infarction, coronary revascularization and ischaemic stroke) per 1.0mmol/L reduction in LDL cholesterol (CTT collaboration 2010). Risk of CVD was increased by 6% in men and 12% in women for each 0.2 mmol/l increase in triglyceride concentrations in a meta-analysis of prospective population studies (Hokanson and Austin 1996).

^6^ The 95%CI does not cross threshold of important benefit or harm.

^7^ Too few studies to reliably assess publication bias (<10 studies).

^8^ The 95%CI crosses zero and does not cross threshold of important benefit or harm and is therefore considered a precise estimate of no effect and not downgraded for serious imprecision.
^9^ The 95%CI may cross threshold of important benefit or harm and is therefore downgraded for serious imprecision.

^10^ The study was conducted in the population of interest, all comparisons were made directly to an appropriate control group and all outcomes were priority outcomes decided upon prior to initiating review. Body weight is a direct measure of growth.

**References** (not cited in article)

Cholesterol Treatment Trialists’ (CTT) Collaboration, Baigent C, Blackwell L, Emberson J, Holland LE, Reith C, Bhala N, Peto R, Barnes EH, Keech A, Simes J, Collins R. Efficacy and safety of more intensive lowering of LDL cholesterol: a meta-analysis of data from 170,000 participants in 26 randomised trials.

Lancet. 2010;376(9753):1670-81.

Hokanson JE, Austin MA. Plasma triglyceride level is a risk factor for cardiovascular disease independent of high-density lipoprotein cholesterol level: a meta-analysis of population-based prospective studies. Journal of cardiovascular risk. Apr 1996;3(2):213-9.

**GRADE evidence profile 4**

**Author(s):** Jason Montez and Lisa Te Morenga
**Question:** What is the effect of replacing some saturated fatty acids in the diet of children with polyunsaturated fatty acids?^1^

**Setting:** General child population

| **Quality assessment** | | | | | | | **No. of participants^2^** | | | **Relative effect (95%CI)** | **Quality** | **Importance** |
| --- | --- | --- | --- | --- | --- | --- | --- | --- | --- | --- | --- | --- |
| **No. of studies** | **Design** | **Risk of bias** | **Inconsistency** | **Indirectness** | **Imprecision** | **Other considerations** | **Reduced saturated fat intake** | | **Usual saturated fat intake** |  |  |  |
| **Total cholesterol (follow-up 5 weeks; units mmol/L; better indicated by lower values)** | | | | | | | | | | | | |
| 1 | RCTs | no serious  risk of bias^3^ | no serious inconsistency^4^ | no serious indirectness^5^ | no serious imprecision^6^ | none^7^ | 134 | | 134 | **MD 0.29 lower**  (0.40 to 0.18 lower) |  HIGH | IMPORTANT |
| **LDL cholesterol (follow-up 5 weeks; units mmol/L; better indicated by lower values)** | | | | | | | | | | | | |
| 1 | RCTs | no serious  risk of bias^3^ | no serious inconsistency^4^ | no serious indirectness^5^ | no serious imprecision^6^ | none^7^ | 134 | | 134 | **MD 0.29 lower**  (0.38 to 0.20 lower) |  HIGH | CRITICAL |
| **HDL cholesterol (follow-up 5 weeks; units mmol/L; better indicated by higher values)** | | | | | | | | | | | | |
| 1 | RCTs | no serious  risk of bias^3^ | no serious inconsistency^4^ | no serious indirectness^5^ | no serious imprecision^8^ | none^7^ | 134 | 134 | | **MD 0.00 lower**  (0.03 lower to 0.03 higher) |  HIGH | IMPORTANT |
| **Serum triglyceride (follow-up 5 weeks; units mmol/L; better indicated by lower values)** | | | | | | | | | | | | |
| 1 | RCTs | no serious  risk of bias^3^ | no serious inconsistency^4^ | no serious indirectness^5^ | serious imprecision^9^ | none^7^ | 134 | 134 | | **MD 0.02 lower**  (0.07 lower to 0.03 higher) |  MODERATE | IMPORTANT |
| **Apolipoprotein A1** | | | | | | | | | | | | |
| 0 | No studies identified reporting this outcome | | | | | | | | | | | |
| **Apolipoprotein B** | | | | | | | | | | | | |
| 0 | No studies identified reporting this outcome | | | | | | | | | | | |
| **Systolic blood pressure** | | | | | | | | | | | | |
| 0 | No studies identified reporting this outcome | | | | | | | | | | | |
| **Diastolic blood pressure** | | | | | | | | | | | | |
| 0 | No studies identified reporting this outcome | | | | | | | | | | | |
| **Body weight (follow-up 5weeks; units kg; better indicated by lower values)** | | | | | | | | | | | | |
| 1 | RCTs | no serious  risk of bias^3^ | no serious inconsistency^4^ | no serious indirectness^10^ | serious imprecision^9^ | none^5^ | 134 | 134 | | **MD 0.20 lower**  (0.63 lower to 0.23 higher) |  MODERATE | IMPORTANT |
| **Height** | | | | | | | | | | | | |
| 0 | No studies identified reporting this outcome | | | | | | | | | | | |
| **BMI** | | | | | | | | | | | | |
| 0 | No studies identified reporting this outcome | | | | | | | | | | | |
| **Waist circumference** | | | | | | | | | | | | |
| 0 | No studies identified reporting this outcome | | | | | | | | | | | |
| **Insulin resistance** | | | | | | | | | | | | |
| 0 | No studies identified reporting this outcome | | | | | | | | | | | |
| **Adverse effects** | | | | | | | | | | | | |
| 0 | No studies identified reporting this outcome | | | | | | | | | | | |

CI, confidence interval; RCTs, randomized controlled trials; MD, mean difference; SMD, standardized mean difference; LDL, low-density lipoprotein; HDL, high-density lipoprotein; BMI, body mass index

^1^ In the included study (Denke 2000), saturated fatty acids (SFA) were replaced almost entirely with polyunsaturated fatty acids (PUFA).

^2^ Participants in this crossover trial are counted in both the reduced and usual SFA intake groups.

^3^ No serious risk of bias in the included study.

^4^ Only one study included.

^5^ The study was conducted in the population of interest, all comparisons were made directly to an appropriate control group and all outcomes are priority outcomes that were decided upon prior to initiating the review. Blood lipids are indirect markers of CVD risk, however, cardiovascular events are rare in children and adolescents. LDL cholesterol is a well-accepted marker of CVD risk in adults: a large meta-analysis of statin therapy RCTs conducted in adults demonstrated a more than 20% reduction in risk of cardiovascular events (coronary death, non-fatal myocardial infarction, coronary revascularization and ischaemic stroke) per 1.0mmol/L reduction in LDL cholesterol (CTT collaboration 2010). Risk of CVD was increased by 6% in men and 12% in women for each 0.2 mmol/l increase in triglyceride concentrations in a meta-analysis of prospective population studies (Hokanson and Austin 1996).

^6^ The 95%CI does not cross threshold of important benefit or harm.

^7^ Too few studies to reliably assess publication bias (<10 studies).

^8^ The 95%CI crosses zero and does not cross threshold of important benefit or harm and is therefore considered a precise estimate of no effect and not downgraded for serious imprecision.
^9^ The 95%CI may cross threshold of important benefit or harm and is therefore downgraded for serious imprecision.

^10^ The study was conducted in the population of interest, all comparisons were made directly to an appropriate control group and all outcomes were priority outcomes decided upon prior to initiating review. Body weight is a direct measure of growth.

**References** (not cited in article)

Cholesterol Treatment Trialists’ (CTT) Collaboration, Baigent C, Blackwell L, Emberson J, Holland LE, Reith C, Bhala N, Peto R, Barnes EH, Keech A, Simes J, Collins R. Efficacy and safety of more intensive lowering of LDL cholesterol: a meta-analysis of data from 170,000 participants in 26 randomised trials.

Lancet. 2010;376(9753):1670-81.

Hokanson JE, Austin MA. Plasma triglyceride level is a risk factor for cardiovascular disease independent of high-density lipoprotein cholesterol level: a meta-analysis of population-based prospective studies. Journal of cardiovascular risk. Apr 1996;3(2):213-9.

**GRADE evidence profile 5**

**Author(s):** Jason Montez and Lisa Te Morenga
**Question:** What is the effect of replacing some saturated fatty acids in the diet of children with monounsaturated fatty acids?^1^

**Setting:** General child population

| **Quality assessment** | | | | | | | | **No. of participants^2^** | | | **Relative effect (95%CI)** | **Quality** | | **Importance** |
| --- | --- | --- | --- | --- | --- | --- | --- | --- | --- | --- | --- | --- | --- | --- |
| **No. of studies** | **Design** | | **Risk of bias** | **Inconsistency** | **Indirectness** | **Imprecision** | **Other considerations** | **Reduced saturated fat intake** | | **Usual saturated fat intake** |  |  |  |  |
| **Total cholesterol (follow-up 7 months; units mmol/L; better indicated by lower values)** | | | | | | | | | | | | | | |
| 1 | RCTs | | no serious  risk of bias^3^ | no serious inconsistency^4^ | no serious indirectness^5^ | no serious imprecision^6^ | none^7^ | 88 | | 88 | **MD 0.33 lower**  (0.52 to 0.14 lower) |  HIGH | | IMPORTANT |
| **LDL cholesterol (follow-up 7 months; units mmol/L; better indicated by lower values)** | | | | | | | | | | | | | | |
| 1 | RCTs | | no serious  risk of bias^3^ | no serious inconsistency^4^ | no serious indirectness^5^ | no serious imprecision^6^ | none^7^ | 88 | | 88 | **MD 0.26 lower**  (0.41 to 0.11 lower) |  HIGH | | CRITICAL |
| **HDL cholesterol (follow-up 7 months; units mmol/L; better indicated by higher values)** | | | | | | | | | | | | | | |
| 1 | RCTs | | no serious  risk of bias^3^ | no serious inconsistency^4^ | no serious indirectness^5^ | serious imprecision^8^ | none^7^ | 88 | 88 | | **MD 0.02 lower**  (0.11 lower to 0.07 higher) |  MODERATE | | IMPORTANT |
| **Serum triglyceride (follow-up 7 months; units mmol/L; better indicated by lower values)** | | | | | | | | | | | | | | |
| 1 | RCTs | | no serious  risk of bias^3^ | no serious inconsistency^4^ | no serious indirectness^5^ | no serious imprecision^6^ | none^7^ | 88 | 88 | | **MD 0.11 lower**  (0.19 to 0.03 lower) |  HIGH | | IMPORTANT |
| **Apolipoprotein A1 (follow-up 7 months; units mg/dL; better indicated by higher values)** | | | | | | | | | | | | | | |
| 1 | RCTs | | no serious  risk of bias^3^ | no serious inconsistency^4^ | no serious indirectness^5^ | serious imprecision^8^ | none^7^ | 88 | 88 | | **MD 2.00 lower**  (8.23 lower to 4.23 higher) |  MODERATE | | IMPORTANT |
| **Apolipoprotein B (follow-up 7 months; units mg/dL; better indicated by lower values)** | | | | | | | | | | | | | | |
| 1 | RCTs | | no serious  risk of bias^3^ | no serious inconsistency^4^ | no serious indirectness^5^ | serious imprecision^8^ | none^7^ | 88 | 88 | | **MD 1.20 lower**  (8.30 lower to 5.90 higher) |  MODERATE | IMPORTANT | |
| **Systolic blood pressure** | | | | | | | | | | | | | | |
| 0 | No studies identified reporting this outcome | | | | | | | | | | | | | |
| **Diastolic blood pressure** | | | | | | | | | | | | | | |
| 0 | No studies identified reporting this outcome | | | | | | | | | | | | | |
| **Body weight** | | | | | | | | | | | | | | |
| 0 | | No studies identified reporting this outcome | | | | | | | | | | | | |
| **Height** | | | | | | | | | | | | | | |
| 0 | | No studies identified reporting this outcome | | | | | | | | | | | | |
| **BMI** | | | | | | | | | | | | | | |
| 0 | | No studies identified reporting this outcome | | | | | | | | | | | | |
| **Waist circumference** | | | | | | | | | | | | | | |
| 0 | | No studies identified reporting this outcome | | | | | | | | | | | | |
| **Insulin resistance** | | | | | | | | | | | | | | |
| 0 | | No studies identified reporting this outcome | | | | | | | | | | | | |
| **Adverse effects** | | | | | | | | | | | | | | |
| 0 | | No studies identified reporting this outcome | | | | | | | | | | | | |

CI, confidence interval; RCTs, randomized controlled trials; MD, mean difference; SMD, standardized mean difference; LDL, low-density lipoprotein; HDL, high-density lipoprotein; BMI, body mass index

^1^ In the included study (Estevez-Gonzalez 2000), saturated fatty acids (SFA) were replaced with a mixture of polyunsaturated fatty acids (20%) and monounsaturated fatty acids (80%).

^2^ Participants in this crossover trial are counted in both the reduced and usual SFA intake groups.

^3^ No serious risk of bias in the included study.

^4^ Only one study included.

^5^ The study was conducted in the population of interest, all comparisons were made directly to an appropriate control group and all outcomes are priority outcomes that were decided upon prior to initiating the review. Blood lipids are indirect markers of CVD risk, however, cardiovascular events are rare in children and adolescents. LDL cholesterol is a well-accepted marker of CVD risk in adults: a large meta-analysis of statin therapy RCTs conducted in adults demonstrated a more than 20% reduction in risk of cardiovascular events (coronary death, non-fatal myocardial infarction, coronary revascularization and ischaemic stroke) per 1.0mmol/L reduction in LDL cholesterol (CTT collaboration 2010). Risk of CVD was increased by 6% in men and 12% in women for each 0.2 mmol/l increase in triglyceride concentrations in a meta-analysis of prospective population studies (Hokanson and Austin 1996).

^6^ The 95%CI does not cross threshold of important benefit or harm.

^7^ Too few studies to reliably assess publication bias (<10 studies).

^8^ The 95%CI may cross threshold of important benefit or harm and is therefore downgraded for serious imprecision.

**References** (not cited in article)

Cholesterol Treatment Trialists’ (CTT) Collaboration, Baigent C, Blackwell L, Emberson J, Holland LE, Reith C, Bhala N, Peto R, Barnes EH, Keech A, Simes J, Collins R. Efficacy and safety of more intensive lowering of LDL cholesterol: a meta-analysis of data from 170,000 participants in 26 randomised trials.

Lancet. 2010;376(9753):1670-81.

Hokanson JE, Austin MA. Plasma triglyceride level is a risk factor for cardiovascular disease independent of high-density lipoprotein cholesterol level: a meta-analysis of population-based prospective studies. Journal of cardiovascular risk. Apr 1996;3(2):213-9.
