# Supplementary figures and images for: Health effects of saturated and trans-fatty acid intake in children and adolescents: Systematic review and meta-analysis
Source: PLoS One. 2017 Nov 17;12(11):e0186672. doi: 10.1371/journal.pone.0186672 (PMC5693282; doi:10.1371/journal.pone.0186672)

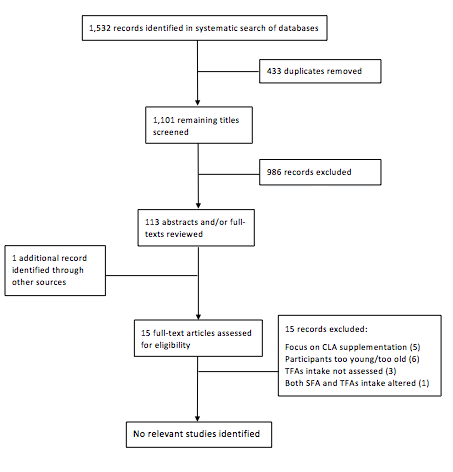

Supplement: S1 Fig — (TIFF) [file pone.0186672.s007.tiff]

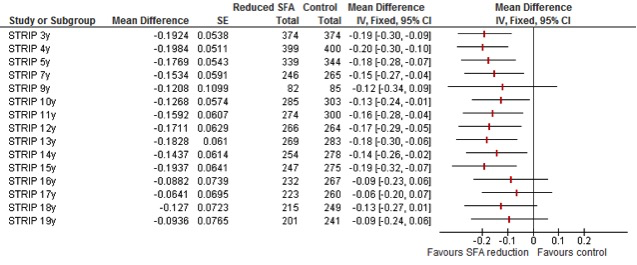

Supplement: S8 Fig — (TIFF) [file pone.0186672.s014.tiff]
